# Rabies healthcare-seeking behaviors of urban and peri-urban residents: Results from a rabies knowledge, attitudes, and practices survey, Bangladesh, 2018

**DOI:** 10.1371/journal.pntd.0010634

**Published:** 2022-08-09

**Authors:** Yasmeen B. Ross, Mahbubul Hoque, Jesse D. Blanton, Erin D. Kennedy, Md Sohel Rana, Sanya Tahmina, Sarah Bonaparte, Jennifer R. Head, Ryan M. Wallace

**Affiliations:** 1 US Centers for Disease Control and Prevention, Atlanta, Georgia, United States of America; 2 Training Programs in Epidemiology and Public Health Interventions Network, Atlanta, Georgia, United States of America; 3 Department of Livestock Services, Ministry of Fisheries and Livestock, Dhaka, Bangladesh; 4 Directorate General of Health Services, Dhaka, Bangladesh; Swiss Tropical and Public Health Institute: Schweizerisches Tropen- und Public Health-Institut, SWITZERLAND

## Abstract

Rabies is one of the most lethal infectious diseases, with those living in Asia and Africa having the highest risk of dying from rabies. We conducted a knowledge, attitudes and practices survey in urban and peri-urban areas of Bangladesh to describe canine bite rates, rabies knowledge, and healthcare seeking behaviors and barriers to human and dog vaccination. A bite risk assessment score (BRAS) and healthcare-seeking behavior score (HSBS) was calculated for each bite victim. Respondents were given two hypothetical situations to assess potential behaviors after a bite and willingness to pay for rabies vaccine and immunoglobulin. In total, 2,447 households participated in the survey and 85 bite victims were identified. The BRAS identified that 31% of bites posed no risk of rabies transmission. Multivariate analyses showed that living in Chittagong (β = 1.4; 95% CI: 0.1, 2.7) was associated with a higher HSBS. Findings presented here provide useful information regarding bite occurrences, healthcare-seeking behaviors, and a need for strategies to increase rabies awareness.

## Author summary

Rabies is one of the most lethal infectious diseases and those living in Asia and Africa have the highest risk of dying from rabies. Bangladesh has the third highest number of estimated human deaths from rabies of all countries with an estimated 2,000–2,500 deaths annually. In 2018, a knowledge, attitude, and practices (KAP) survey was conducted to better describe the rabies burden in four urban and rural areas. Our manuscript will deepen the understanding of ways to develop cost-effective, risk-based PEP delivery to persons with probable rabies exposures to prevent unnecessary PEP shortages among urban and peri-urban populations. The findings here provide useful information regarding bite occurrences, healthcare-seeking behaviors, and a need for strategies to increase rabies awareness in urban and peri-urban areas. This study confirmed a high human rabies death rate in Bangladesh despite a high rate of PEP usage and completion rates and makes a case for IBCM as cost effective means to rabies control in Bangladesh.

## 1. Introduction

Rabies is one of the most lethal infectious diseases with the highest human case fatality rate of all conventional etiological agents [1]. Rabies virus is responsible for an estimated 59,000 human deaths each year; bites from rabid dogs (i.e. dog-mediated rabies) are responsible for causing over 95% of human rabies deaths [2,3]. Over 70% of the world’s population resides in areas where dogs are a reservoir for rabies [4].

Those living in Asia and Africa have the highest risk of dying from rabies. Approximately 22,000–28,000 human rabies deaths occur annually in the World Health Organization (WHO) South East Asia Region (SEARO), accounting for 45% of annual global human rabies deaths [5,6]. The economic burden of rabies in this region is estimated to be 563 million U.S. dollars annually [1]. Since dogs are responsible for nearly all human rabies deaths in SEARO, control of rabies within dog populations is the prioritized strategy in preventing human rabies deaths there [5,6]. Given the rabies burden in the region, the eight member countries of the South Asian Association for Regional Cooperation (SAARC), including Afghanistan, Bangladesh, Bhutan, India, the Maldives, Nepal, Pakistan, and Sri Lanka, identified rabies control as a priority. Collectively, they created the ‘SAARC Rabies Elimination Project’ to develop a regional platform for the elimination of dog-mediated rabies [5,6].

Globally, Bangladesh has the third highest number of estimated human deaths from rabies of all countries, with an estimated 2,000–2,500 deaths annually [7]. As a member of the SAARC, Bangladesh has declared the elimination of dog-mediated human rabies deaths by 2030 as a national goal. This strategy for elimination is focused on dog vaccination, bite prevention education programs, establishment of a rabies surveillance system in domestic and wild animals, and prompt post-exposure prophylaxis (PEP) with rabies immunoglobulin (RIG) for humans with exposures to suspected rabid animals [5]

In Bangladesh, over 250,000 people received rabies PEP in 2010, access to PEP has improved over the last decade [8]. Bangladesh’s current rabies PEP regimen for previously unimmunized persons follows the updated Thai Red Cross schedule which recommends two site intra-dermal administration of rabies vaccine on days 0, 3, 7, 28 and additional doses of rabies immunoglobulin (RIG) for category III exposures (e.g. transdermal bite or scratches) as defined by the WHO [1]. There are 66 public District Rabies Prevention and Control Centers (DRPCCs) and one National Rabies Prevention and Control Center (NRPCC) within the 64 districts of Bangladesh where bite victims can receive free rabies vaccination and RIG. Victims are only charged the cost of disposable syringes ($0.13 USD/11.05 BDT). On average, the NRPCC administers rabies PEP to approximately 500 patients daily [9]. Since 2014, the Communicable Disease Control Operational plan of the Directorate General of Health Services (CDC/DGHS, Bangladesh) has annually increased their vaccine supply by 20–30% to meet the demand at NRPCC and by 50–80% to meet the demand at DRPCCs. Despite these increases in government-procured human rabies vaccine supply, local and national shortages are reported each year [9].

While the current approach to dog bite management provides virtually free rabies vaccination to residents when available and has reduced the human rabies burden in Bangladesh, the approach is not without weaknesses. First, previous studies found that low rabies awareness and PEP use in Southern Asia are likely barriers to the elimination of dog-mediated human rabies deaths [10,11]. A study in eight Asian countries, including Bangladesh, China, India, Indonesia, Pakistan, the Philippines, Sri Lanka, and Thailand, found that among patients who reported bite exposures, 34% did not know about rabies before being bitten, 30% did not know where to find rabies prevention centers, 50% sought advice from a medical doctor, and only 22% received RIG (despite being recommended for 43% of patients) [10]. A different knowledge, attitudes and practices (KAP) survey also indicated low rabies knowledge, with 58% of respondents being unaware of the consequences of a dog bite. A survey of residents from Satkhira Sadar, a southwestern sub-district of Bangladesh, reported higher (>73%) awareness of rabies, its causes, and prevention measures was reported in the aforementioned study [11]. However, only 29% of bite victims received rabies vaccination and none received RIG in that survey study. Second, when the demand for the vaccine exceeds the supply of the subsidized vaccine, patients must purchase vaccine and RIG from pharmacies at a cost of $8.25 USD (701.04 BDT) and $12.50 USD (1062.19 BDT) per vial, respectively [9].

Animal rabies control programs are limited in Bangladesh, leading to a high risk for the transmission of rabies from biting dogs and a low capacity for veterinary response to reports of animal rabies exposure events. Accordingly, any animal bite is currently considered to be a potentially suspect rabies exposure. In contrast, Integrated Bite Case Management (IBCM) is an approach that links public health professionals and veterinarians to assess whether or not a dog bite is considered to be a likely rabies exposure [12]. The WHO and other global partners now emphasize dog vaccination and PEP provision conditional on IBCM-based risk assessments in lieu of a model where everyone with a recent bite receives PEP. Such techniques can reduce unnecessary use of PEP, improving cost-effectiveness of rabies control programs [13].

A 2012 study evaluated animal bites and rabies in humans among rural communities in Bangladesh [14]. The study focused only on rural sub-districts and estimated the number of bites and healthcare-seeking behaviors of bite victims using a snowball sampling technique. In this study, we explored rabies knowledge, attitudes towards dog bites, and practices of bite victims in urban and peri-urban settings in Bangladesh. The objective of this study was to: 1) estimate canine bite rates in urban and peri-urban settings in Bangladesh; 2) assess knowledge of rabies risks and protective measures; 3) evaluate health care seeking behaviors and barriers to human and dog vaccination. Ultimately, the results of the survey will deepen understanding of ways to develop cost-effective, risk-based PEP delivery to persons with probable rabies exposures to prevent unnecessary PEP shortages among urban and peri-urban populations.

## 2. Methods

### 2.1 Ethics statement

At each household, a voluntary verbal informed consent was obtained from each interviewee as well as the GPS coordinate of the household to validate study area. No names or other directly identifiable information were collected as a part of this survey. This study protocol was developed in collaboration with Bangladesh Directorate General of Health Services and was approved by the Centers for Disease Control Human Research Protection Office under Protocol ID 060118JB.

### 2.2 Survey and study population

A cross-sectional household survey was conducted in four districts of Bangladesh, two of which represented urban areas and two peri-urban areas, using a stratified, multi-stage cluster design with random sampling. Survey sites were selected by Ministry of Health officials to coincide with city corporations and peri-urban upazillas (sub-districts) randomly where canine vaccination campaigns were being conducted. A structured questionnaire with Bengali translation was modified from questionnaires previously conducted in other countries to collect data through a face-to-face interview with participants in the selected sites [15,16]. Knowledge, attitudes, and practices (KAP) survey questions collected information pertaining to (i) demographics, (ii) dog bite rates, (iii) attitudes about dog bites and dog ownership, (iv) willingness to pay for human rabies vaccination, (v) healthcare access knowledge, (vi) rabies disease and vaccination knowledge, (vii) dog ownership practices, (viii) biting dog health status, and (ix) campaign awareness and barriers to rabies vaccinations for dogs (S1 Text). Interviewers used cell phones with the digital data collection tool KoBoCollect installed to record responses (Harvard Humanitarian Initiative, Cambridge, Massachusetts, USA). Interviewers selected by ministry officials were community health workers fluent in English and Bengali with prior experience conducting interviews. Prior to the survey, interviewers participated in a 2-day training held by the US Centers for Disease Control (US-CDC), Bangladesh’s Department of Livestock Services (DLS), and Directorate General of Health Services (DGHS).

**Fig 1:**
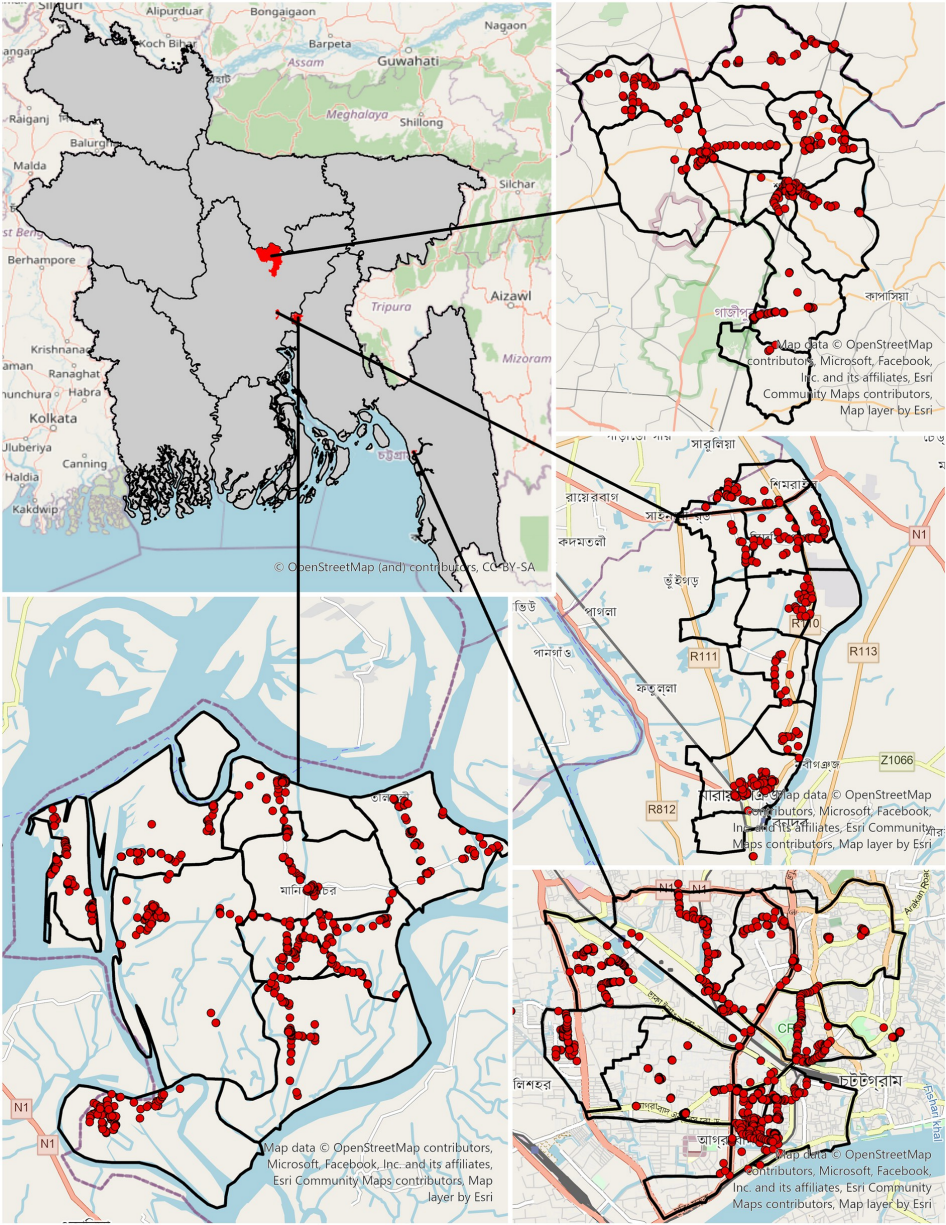
Map of Bangladesh survey sites and the distribution of household surveys by survey site. (A) Map of country with selected evaluation sites highlighted in red (B) Sreepur study site with distribution of surveys. (C) Narayanganj study site with distribution of surveys. (D) Chittagong study site with distribution of surveys. (E) Meghna study site with distribution of surveys. Base map data from OpenStreetMap (https://cdn.arcgis.com/sharing/rest/content/items/3e1a00aeae81496587988075fe529f71/resources/styles/root.json). Administrative boundaries from Humanitarian Data Exchange (Bangladesh - Subnational Administrative Boundaries - Humanitarian Data Exchange (humdata.org).

Two divisions, Dhaka and Chittagong, were selected for survey administration. From these divisions, the districts Chittagong (Chittagong Division) and Narayanganj (Dhaka Division) were selected to represent urban areas, and Sreepur (Dhaka Division) and Meghna (Chittagong Division) were selected to represent peri-urban areas (Fig 1). The first household was selected as the central point of the respective area (e.g. zero-point, cross street, or largest structure, etc.) and the direction was determined by the spinning of a pen. In urban areas every 40^th^ household was interviewed and in the peri-urban area every 16^th^ household was interviewed. If the interviewer reached an apartment building, each apartment in the building was counted as a household unit (counting began in the apartments from the ground floor). If the selected household was empty or when no one was home, the next closest household was selected for interview. Reaching any intersection, spinning of a pen indicated the next direction. If the interviewer came at the end of the assigned zone, the spinning pen directed the new direction of the survey after backtracking to the central point. One member of the household, who was either the head of household or met the inclusion criteria when the head of household was unavailable, was interviewed. Inclusion criteria for survey participation were: 18 years or older, a member of the selected household, and willing and able to provide informed consent. Information regarding dog bite occurrences and following healthcare-seeking behaviors was reported retrospectively by the survey respondent for all household members who had known dog bites within the 12 months prior to the survey date, even if the household member was no longer alive. Interviewees also provided responses regarding their knowledge of rabies and willingness to pay for rabies vaccination. The demographics of surveyed household respondents were compared by sub-district (Table 1). Our sample size was calculated for each field site based on an expected bite rate of 5% using the cohort methodology described by Fleiss et al with continuity correction, resulting in a sample size of approximately 660 surveys per field site, or 2,640 for the full study [17].

**Table 1:** Demographics of Rabies Knowledge, Attitudes, and Practices survey respondents, Bangladesh 2018.

	All Households	Peri-urban		Urban	
		Meghna^a^ (Chittagong Division)	Sreepur^a^ (Dhakka Division)	Chittagong^a^ (Chittagong Division)	Narayanganj^a^ (Dhakka Division)
	n (%)	n (%)	n (%)	n (%)	n (%)
**Gender**					
Female	746 (30%)	168 (30%)	258 (37%)	200 (34%)	120 (20%)
Male	1701 (70%)	399 (70%)	446 (63%)	383 (66%)	473 (80%)
**Age (years)**					
18–30	706 (29%)	169 (30%)	247 (35%)	190 (33%)	100 (17%)
31–40	446 (18%)	97 (17%)	131 (19%)	110 (19%)	108 (18%)
41–50	408 (17%)	77 (14%)	88 (13%)	84 (14%)	159 (27%)
51–79	342 (14%)	91 (16%)	97 (14%)	56 (10%)	98 (17%)
80+	8 (0.3%)	1 (0.2%)	5 (1%)	0 (0%)	2 (0.3%)
**Years of schooling completed by any member of household**					
No education	362 (15%)	79 (14%)	122 (17%)	98 (17%)	63 (11%)
Primary	484 (20%)	163 (29%)	120 (17%)	91 (16%)	110 (19%)
Secondary	1479 (60%)	319 (56%)	444 (63%)	333 (57%)	383 (65%)
Degree	132 (5%)	11 (2%)	33 (5%)	55 (9%)	33 (6%)
Masters and above	51 (2%)	6 (1%)	11 (2%)	20 (3%)	14 (2%)
**Religion**					
Hinduism	138 (6%)	17 (3%)	21 (3%)	66 (11%)	34 (6%)
Islam	2297 (94%)	550 (97%)	679 (96%)	511 (88%)	557 (94%)
Other	12 (1%)	0 (0%)	4 (0.6%)	6 (1%)	2 (0.3%)
**Dog ownership**					
Dog owning households	126 (5%)	43 (8%)	30 (4%)	14 (2%)	39 (7%)
Number of Dogs Owned	183	57	42	22	62
Dogs per Dog Owning HH	1.5 (1.2–1.7)	1.3 (1.0–1.7)	1.4 (1.0–1.9)	1.6 (1.0–2.3)	1.6 (1.2–2.0)
**Total Household Population**	13529 (1%)	2984 (2%)	3830 (1%)	3283 (1%)	3432 (1%)
**Households Interviewed**	2447	567	704	583	593
**Population Estimate**	1370600	125513	321454	470456	453177
**Bite rate (per 100 people)**	0.6 (0.51–0.77)	0.4 (0.19–0.64)	1.1 (0.8–1.47)	0.5 (0.27–0.74)	0.5 (0.3–0.78)
**Average Distance to a PEP Center (km)**	7.6 (7.4–7.7)	9.2 (8.9–9.4)	10.9 (10.62–11.1)	4 (3.8–4.2)	5.6 (5.4–5.8)
**Wealth Score** ^**b**^	77.2 (76.8–77.5)	71.5 (70.8–72.2)	68.9 (68.3–69.5)	86 (85.3–86.8)	83.7 (83.0–84.5)
**Knowledge Score** ^**c**^	33.1 (32.8–33.3)	28.5 (28.1–29.0)	32 (31.6–32.5)	23.4 (23.0–23.8)	48.1 (47.6–48.7)

^a^ Urban and peri-urban sub-districts were selected from Chittagong and Dhakka divisions.

^b^ Wealth score is a composite score based on scoring of four variables: (i) owned household items, (ii) type of toilet facilities, (iii) source of drinking water, and (iv) household wall construction.

^c^ Knowledge score based upon responses to five rabies knowledge questions among respondents that acknowledged that they had heard of the disease “rabies”.

An annual dog bite rate per 100 residents was calculated for each sub-district. Characteristics of the biting dog were also assessed; dog ownership status was compared to the age of the bite victim and dog survival.

### 2.3 Household wealth and rabies knowledge

We calculate a composite wealth score and rabies knowledge score for each household based on responses to select survey questions. Following previous work, we calculated the composite wealth scores based on scoring of four variables: (i) owned household items, (ii) type of toilet facilities, (iii) source of drinking water, and (iv) household wall construction (Box 1) [18]. Household items were assigned an ordinal point value of one to five, based upon their relative monetary worth. We summed values of household items and then standardized to a scale of zero to one. The factors related to sanitation, type of drinking water, and complexity of toilet construction were evaluated based on their reflection of economic status on a scale of 0 (less advanced toilet construction / drinking water from pond, river, lake) to 1 (advanced toilet construction / drinking water piped inside). Housing construction materials were assessed by the trained surveyor; households with higher quality construction material, such as brick, were given a score of one. The final wealth score was derived from an equally weighted sum of the four variables, standardized to a scale of 0 to100. For respondents who did not provide a response, a score was imputed from the average of the district.

**Box 1. Wealth Score Calculation**

**Table:** 

Household Item	N	%	Points Assigned	Summarized score
Electric Fan	2332	94.18%	1	0.07
Television	2306	93.13%	2	0.13
Refrigerator	1759	71.04%	3	0.20
Electricity	2205	89.05%	4	0.27
Bank Account	676	27.30%	5	0.33
Highest Score			15	
**Toilet Facility**	**N**	**%**	**Points Assigned**	**Summarized score**
No facility / bush / field	12	0.48%	0	0.00
Hanging Latrine	55	2.22%	1	0.20
Pit Latrine	106	4.28%	2	0.40
Pucka Toilets	25	1.01%	3	0.60
Flush—to open latrine	381	15.39%	4	0.80
Flush—to sewer or tank	1886	76.17%	5	1.00
I do not know	9			
Highest Score			5	
**Drinking Water**	**N**	**%**	**Points Assigned**	**Summarized score**
Pond/River/Lake Water	13	0.53%	0	0.00
Dug well	8	0.32%	1	0.33
Tube well	976	39.42%	2	0.67
Piped inside	1474	59.53%	3	1.00
No response/Other	5			
Highest score			3	
**Wall Material**	**N**	**%**	**Points Assigned**	**Summarized score**
Cane/palm/trunks	12	0.48%	0	0.00
Dirt / Mud	353	14.26%	1	0.25
Tin	796	32.15%	2	0.50
Cement	741	29.93%	3	0.75
Brick	565	22.82%	4	1.00
No response/Other	9			
Highest score			4	

N = the frequency of each response. For household items, more than one item was able to be selected.

We calculated rabies knowledge scores based upon responses to five rabies knowledge questions among respondents that acknowledged that they had heard of the disease “rabies” (Box 2). Those who had not heard of rabies received a default score of zero and were not asked the five rabies knowledge questions. Correct responses received 20 points, those who indicated that they did not know the answer received 10 points, and incorrect answers received zero points to account for potentially harmful outcomes based on incorrect knowledge. The highest possible score a respondent could receive was 100. Responses that were excluded in the final dataset include those who did not respond to the question about prior knowledge of rabies and those who had heard of rabies but did not provide responses to three or more questions.

**Box 2. Knowledge Score Calculation**

**Table:** 

Question	First preferred answer (points)	Unreported answers (points)	Incorrect answers (points)
How severe is the disease called ’rabies’?	Very severe, fatal (20)	Decline to answer (10) Blank (10)	Mild (0) Somewhat severe (0) Very severe, but recoverable (0) Don’t know (0)
How do humans get rabies from an infected animal?	Bite (20) Scratch (20) Contact with saliva (20)	Decline to answer (10) Blank (10)	Observing the animal (0) Touching the animal (0) Contact with blood (0) Contact with urine/feces (0) Don’t know (0)
For each animal, what is the risk of that animal having rabies on a scale of 1 to 5, with 1 being little to no risk and 5 being the highest risk?	Dogs, 4–5 (20)	Blank (10)	Dogs, 1–3 (0)
For each animal, what is the risk of that animal having rabies on a scale of 1 to 5, with 1 being little to no risk and 5 being the highest risk?	Rodents, 1–2 (20)	Blank (10)	Rodents, 3–5 (0)
How often should a dog be vaccinated against rabies?	At least once a year (20)	Blank (10)	Every other year (0) 2–3 times per lifetime (0) Once in a lifetime (0) Don’t know (0)

## 2.4 Bite risk and health behaviors

We also calculated a composite bite risk assessment score (BRAS) for each reported dog bite victim to approximate the WHO-recommended post-bite rabies risk assessment criteria and associated PEP recommendations [1]. If the dog was alive 10 days following the bite, the bite was automatically considered no risk. If the dog died within the 10 days following the bite, the bite was automatically considered high risk. If the health status of the dog after 10 days in unknown, we used the anatomical bite location, the bite severity, and the familiarity of the biting dog to classify the risk as “low”, “moderate” or “high” (Box 3). For anatomical bite location, locations such as hands and feet were considered low risk and locations such as the head considered high risk [1, 19]. In cases where the dog bit the victim in more than one anatomical location, the highest risk anatomical location was used in the final calculation. Bite severity is based upon the number of anatomical bite locations. Bite location and bite severity variables were scored one (low risk), two (moderate risk), or three (high risk) while familiarity with the dog was scored zero (no risk–dog owned by the family), one (low risk–known, owned dog), two (moderate risk–known, stray dog), or three (high risk–unknown dog). The final bite risk score for those bitten by a dog with an unknown 10-day health outcome was derived from an equally weighted average of the three BRAS variables adjusted to a scale of 0 to 100 and categorized as low (0–33), medium (34–66), or high (>66). The BRAS in conjunction with the bite rate was also used to estimate the cost of initiating rabies PEP if the cost for vaccine and syringe is $2.19 USD (187.95 BDT) and the cost for 1 vial of RIG and syringe is $12.63 USD (1,083.94 BDT). This assumes that only the wound is infiltrated according to WHO recommendations [9, 20].

**Box 3. Bite Risk Assessment Score (BRAS)**

**Table:** 

Points Assigned	Bite Location(a)	Bite Severity	Familiarity with Dog	Did the animal die within 10 days?
0			Own dog	No
1	Leg/Foot	Single	Neighbor’s dog	
2	Chest/Arm/Hand	Not reported	I don’t know this dog	I don’t know
3	Head/Neck	Multiple	Unowned dog	Yes

BRAS score calculated for each bite based on the respondent’s recollection of the bite event(s). Points assigned based on four indicators. (a) for bites in multiple locations, the most severe bite was used to calculate the BRAS score

Health-seeking behavior score (HSBS) was calculated to grade post-exposure healthcare-seeking decisions for dog bite victims. The HSBS is a composite value of preferred actions recommended by WHO after a bite: washing of the wound, seeking medical care, initiating PEP, completing PEP, and receiving RIG. Bite victims were awarded one point each for indicating each of these behaviors, zero for not performing the behavior, or a half-point for missing responses, resulting in a range of zero to five for this composite variable.

We also presented respondents with two hypothetical situations to assess potential behaviors after a bite. Respondents were asked what post-exposure healthcare-seeking behaviors they would exhibit in hypothetical scenarios where 1) they were bit by a dog they recognized, and 2) they were bit by a dog they did not recognize (S1 Text).

### 2.5 Willingness to pay for RIG and PEP

**Fig 2:**
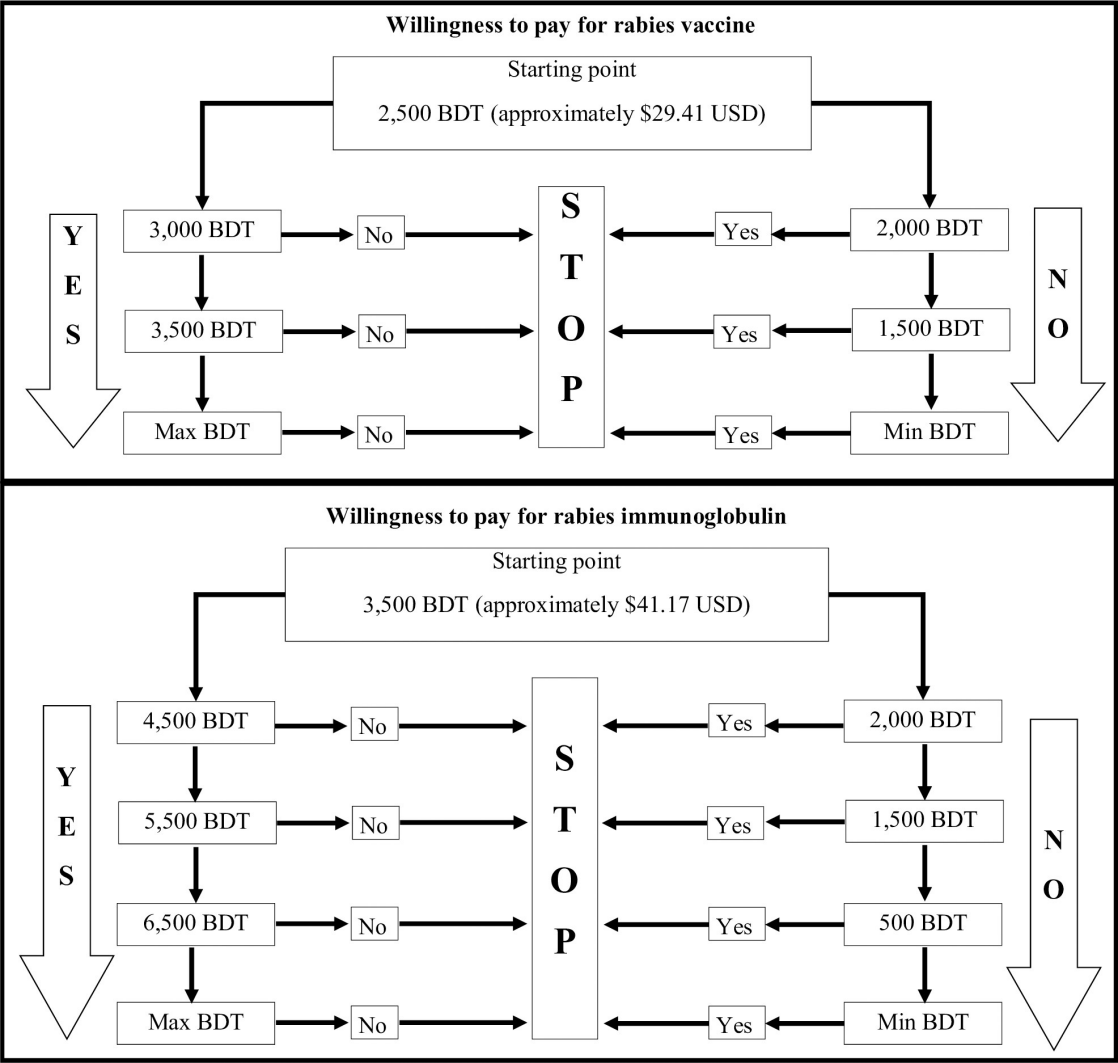
Structure of ‘biding’ game used to determine willingness to pay for rabies vaccine and RIG; RIG = rabies immunoglobulin protein.

We assessed the willingness to pay for rabies vaccine and immunoglobulin based on responses to questions regarding a hypothetical dog bite to the leg, following methods described by Birhane et. al [21]. Respondents were first asked how much they were willing to pay for rabies vaccine starting from 2,500 Bangladeshi Taka (BDT), which is equivalent to approximately 22% of a monthly household income (Fig 2) [22]. Based on their response to this starting value, respondents were then asked willingness to pay at increments, or ‘bids,’ of 500 BDT in the positive or negative direction. If respondents did not provide a final minimum or maximum value within 3 bids, they were asked to specify a value. Subsequent to the vaccine cost bid, they were asked how much in addition to the previously stated amount they were willing to pay for rabies immunoglobulin (RIG), starting from 3,500 BDT and increasing or decreasing in increments of 1,000 BDT. The maximum amount that respondents were willing to pay for vaccine and RIG were combined for the maximum amount willing to pay for PEP. Willingness to pay was compared between respondents’ wealth and rabies knowledge. Interviewees who chose not to respond to both questions regarding vaccine and RIG were excluded from this sub-analysis.

### 2.6 Statistical methods

Statistical analyses were performed using Microsoft Excel (Microsoft Corporation, Redmond, Washington, USA), OpenEpi (http://www.openepi.com/), and SAS v9.4 (SAS Institute, Cary, North Carolina, USA). Chi-square tests were used to compare associations between categorical variables and t-tests were used to compare the means of continuous variables. A cost analysis was conducted based on PEP usage by bite victims and reported cost of biologics detailed in Li et. Al, 2019.

#### 2.6.1 Attitudes

Respondents’ attitudes regarding rabies post-exposure healthcare-seeking behaviors were assessed as well as the influence of recognition of the biting dog on these behaviors. Survey proportions were used to estimate rates of dog bite victims employing individual healthcare-seeking behaviors. We calculated risk ratios, representing the probability of a particular healthcare seeking behavior following a bite from a known dog to its probability following a bite from a dog they do not recognize. We assessed whether the difference in behavior differed significantly by whether or not the dog was known using a chi-squared test, with p-values ≤0.05 considered statistically significant.

#### 2.6.2 Practices

Factors associated with favorable post-exposure healthcare seeking decisions among dog bite victims (n = 85) were assessed using bivariate and multivariate linear regression. We regressed the bite victims’ healthcare seeking behavior scores (HSBS) against multiple independent factors collected through the KAP survey. Independent variables of interest for these analyses included age of the bite victims, highest education level of a household member, cost of travel to the nearest location where rabies PEP could be received and ownership status of the biting dog. To categorize risk the status of the biting dog 10 days after exposure and anatomical location of the bite on the victim were also included. All variables were considered epidemiologically important to adjust for in the multivariable analysis as they were considered to be associated with the other independent variables of interest in the population, causal of the outcome, and not on the causal pathway between the independent variables and the outcome. District was included as an independent variable to account for the clustering of data within the different survey settings. Beta estimates reflecting the mean differences in HSBSs among those with and without the factor of interest, with corresponding 95% confidence intervals, were calculated for each independent variable, and p-values ≤0.05 were considered to represent a statistically significant association.

## 3. Results

### 3.1 Survey and study population

**Fig 3:**
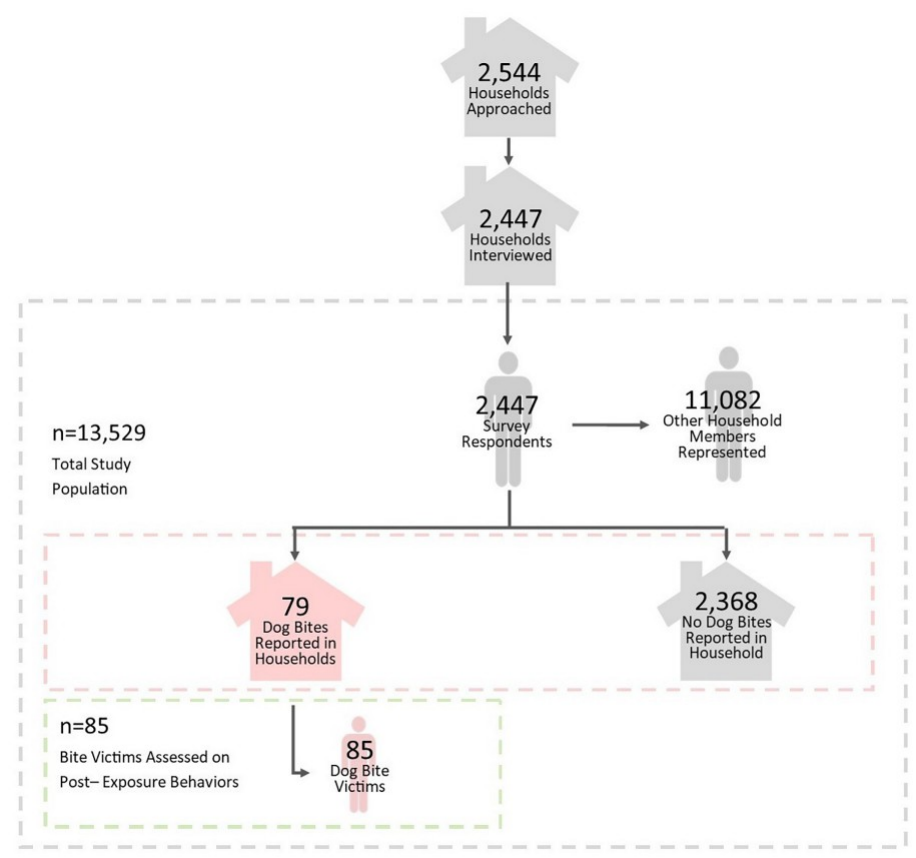
Study populations identified by rabies knowledge, attitudes, and practices survey, Bangladesh 2018.

Interviewers approached 2,544 households and completed surveys at 2,447 households representing 13,529 household members, 85 of whom had experienced a dog bite within one year prior to survey administration (Fig 3). Survey proportions were translated into population rates using 2017 population estimates from the CIA World Factbook (Table 1) [23].

Most respondents interviewed were male (70%) and the largest proportion of all respondents were between the ages of 18 and 30 (29%) (Table 1). A secondary education was the highest educational level of any household member for 60% of surveyed households. While the average wealth score of a household was 77.2 out of 100, wealth scores of peri-urban households were lower than those of urban households (70.05 vs 84.87, p-value <0. 001 respectively). Overall, rabies knowledge scores were low, with the average score among all surveyed households being 33.1 out of 100. In urban areas, the average knowledge score was 35.8 out of 100 while the average knowledge score in peri-urban areas was 30.5 (p-value <0.001). Among respondents, 47% indicated that they were not familiar with rabies disease prior to this survey. Both Chittagong and Meghna, located in the Chittagong division, had the lowest scores for rabies knowledge of the four districts compared with Sreepur and Narayanganj, with means of 28.5 and 23.4 compared to 32.0 and 48.1, respectively (p-value <0.001).

### 3.2 Healthcare-seeking behaviors of bite victims

The survey identified 85 dog bite victims resulting in an overall annual bite rate of 628 (95% CI 505–773) per 100,000 residents (Table 1). We found that the largest proportion of the dog bite victims were less than 14 years old (44%), located in Sreepur (49%), experienced a bite to the arm or hand (89%), and was bitten by a recognized, unowned community dog (61%) (Table 2). Based on the calculated bite risk assessment scores (BRASs), 31% of bites posed no risk of rabies transmission, 24% were low risk, 34% were moderate risk, and 12% were high risk. The health status 10 days after a bite for unowned dogs was not known in 67% of the reported bites (Table 3).

**Table 2:** Characteristics of 85 bite victims, Rabies Knowledge, Attitudes, and Practices Survey, Bangladesh 2018.

	All Bite Victims N = 85	Population Adjusted		
	n (%)	n^a^	Rate per 100,000 people	95% CI
**Age**				
0–14	37 (44%)	3,734	990.9	707.9–1351.0
15–24	21 (25%)	2,613	803.7	501.8–1,208.0
25–54	22 (26%)	5,364	410.1	263.6–610.8
>55	5 (6%)	1,776	281.5	103.2–624.0
**Location**				
Chittagong	15 (18%)	3,283	456.9	265.5–736.7
Meghna	11 (13%)	2,984	368.6	193.8–640.7
Narayanganj	17 (20%)	3,432	495.3	298.2–777.0
Sreepur	42 (49%)	3,830	1,096.6	800.5–1,468.0
**Ownership of Biting Dog**				
Owned	4 (5%)	13,529	29.6	9.4–71.3
Neighbors	8 (9%)	13,529	59.1	27.5–112.3
Recognized Community Dog	52 (61%)	13,529	384.4	290–500.1
Other/Unknown	21 (25%)	13,529	155.2	98.7–233.2
**Bite Location** *				
Head/Neck	4 (5%)	13,529	29.6	9.4–71.3
Chest	3 (4%)	13,529	22.2	5.6–60.4
Arm/Hand	76 (89%)	13,529	561.8	445.7–699.2
Leg/Foot	7 (8%)	13,529	51.7	22.6–102.3
**Bite Risk**				
No Risk	26 (31%)	13,529	192.2	128.2–277.6
Low	20 (24%)	13,529	147.8	92.8–224.3
Moderate	29 (34%)	13,529	214.4	146.3–303.8
High	10 (12%)	13,529	73.9	37.5–131.8

^a^ Population adjusted by age structure and location of Bangladesh. (https://www.cia.gov/the-world-factbook/countries/bangladesh/#people-and-society)

*Multiple selections possible

**Table 3:** Outcomes of biting dogs by ownership status, Rabies Knowledge, Attitudes, and Practices Survey, Bangladesh, 2018.

	Biting Dog Ownership Status				
	Owned by Bite Victim	Owned by Neighbor of Bite Victim	Known, Community Dog	Unrecognized Dog	TOTAL
**Health Status of Biting Dog 10 Days Post-Exposure**					
Known to Have Passed Quarantine	1 (25.0%)	5 (62.5%)	16 (31.4%)	4 (19.0%)	26 (30.6%)
Died during Quarantine	2 (50.0%)	0 (0.0%)	4 (7.8%)	3 (14.3%)	9 (10.6%)
Unknown Health Outcome	1 (25.0%)	3 (37.5%)	32 (61.5%)	14 (66.7%)	50 (58.8%)
**Age Category of Bite Victims**					
0–14	2 (50%)	1 (12.5%)	27 (51.9%)	7 (33.3%)	37 (43.5%)
15–24	2 (50%)	4 (50.0%)	8 (15.4%)	7 (33.3%)	21 (24.7%)
25–54	0 (0.0%)	2 (25.0%)	14 (26.9%)	6 (28.6%)	22 (25.9%)
55–64	0 (0.0%)	1 (12.5%)	2 (3.9%)	1 (4.8%)	4 (4.8%)
65+	0 (0.0%)	0 (0.0%)	1 (1.9%)	0 (0.0%)	1 (1.2%)
**TOTAL**	4	8	52	21	

**Fig 4:**
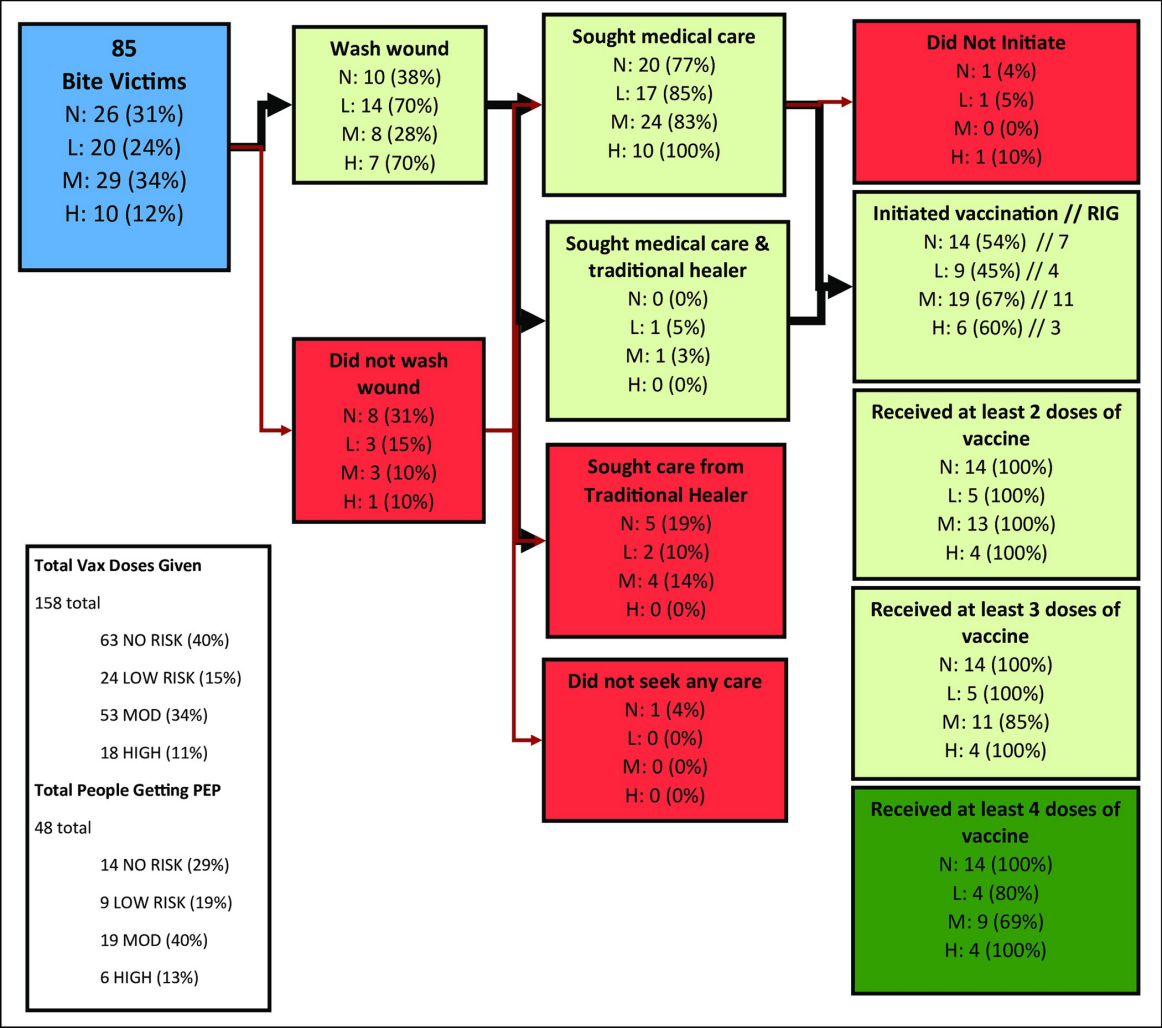
Healthcare-seeking behaviors of bite victims, Rabies Knowledge, Attitudes, and Practices Survey, Bangladesh, 2018. Percentages for post bite behaviors are calculated by risk category. Percentages for vaccine compliance is determined by the number of bite victims who initiated vaccination. N: no risk; L: low risk; M: moderate risk; H: high risk. Risk categories are defined according to the bite risk assessment score, which considers dog familiarity, dog health status and bite location and severity. Percentages may not equal 100% due to rounding. Green boxes indicate favorable healthcare seeking behaviors and red represent unfavorable healthcare seeking behaviors.

Overall, 46% of the 85 bite victims washed the wound and 56% initiated PEP. Of those who initiated PEP 36% received the recommended 4 doses and 52% received rabies immunoglobulin (RIG) (Fig 4). Of the 26 bite victims with no risk of rabies transmission, 38% washed the wound and 54% initiated PEP with a 100% completion rate of the PEP schedule. Initiation of PEP did not appear to differ with bite risk. Among those whose dog bite was considered low risk of rabies transmission, 70% washed the wound and 45% initiated PEP with an 80% completion rate of the PEP schedule. Of those in the moderate risk category, 28% washed the wound and 67% initiated PEP with a 69% completion rate of the PEP schedule. Of the bite victims in the high-risk category, 70% washed the wound and 60% initiated vaccination with a PEP schedule completion rate of 100%. Based on the age-adjusted rate of reported PEP initiation in Bangladesh, 584,006 people initiate the series each year. Based on the bite risk assessment (BRAS) among this population, we estimate that 181,042 (31%) annual bite victims are at no rabies risk, yet still receive vaccination.

We identified three bite victims who sought medical care but did not go on to receive PEP. Using the BRAS, we determined the risk of these three bites to be one each of no risk, low risk, and high risk. When asked why these bite victims did not receive PEP responses included; the vaccine was too expensive (no risk bite victim), they did not feel it was necessary (low risk bite victim), and the vaccine was not available (high risk bite victim). All three bite victims were alive at the time of this survey with bites occurring within 1 month, 9 months, and 12 months prior to the survey, respectively.

Another three bite victims were reported to have died or were not able to be located prior to the time of survey administration. Deceased bite victim number one was 10 years of age and was bitten on the arm/hand by an unowned dog. This dog was reported to have been healthy after 10 days (no risk bite), so this death is not likely due to rabies from this dog bite. The second bite victim, presumed deceased as respondent could not confirm they were alive, was 12 years of age and was bitten in the arm/hand by an unowned dog. The health status of the dog after 10 days was unknown, and this bite was classified as being a moderate risk for rabies. While this person did not seek medical care or PEP, they did see a traditional healer. The last deceased bite victim was 1 year old. This individual was bitten by their family dog in the head/neck area. This dog died within the 10 days resulting in this being considered a high-risk bite occurrence. It was unknown to the respondent if the child’s wound was washed. While medical care was sought and 3 doses of PEP were administered, there was a 16-day delay in seeking care. No additional information pertaining to cause or time of death and clinical signs and symptoms were collected during this survey. The resulting human rabies death rate among this survey population was 14.8 probable deaths per 100,000 population (2.5–48.8 per 100,000).

Using the cost of rabies biologics, we calculate that an estimated $214,345 USD (18,395,583.04 BDT) is spent on the initiation of rabies vaccination and $1,143,297 USD (98,120,389.56 BDT) on RIG for individuals at no risk. Total expected expenditures for rabies PEP in Bangladesh are $4,530,717 USD (388,836,598.90 BDT), of which 31% is spent on persons with no risk for a rabies exposure.

In bivariate analyses, being bitten on the chest/torso (β = 1.98; 95% CI: 0.38, 3.57), living in Chittagong (β = 0.94; 95% CI: 0.17, 1.71), and living 6–20 km from where rabies PEP could be received (β = 0.73; 95% CI: 0.03, 1.43) were positively and significantly associated with a higher HSBS among dog bite victims (Table 4). In multivariate analyses, only living in Chittagong (β = 1.30; 95% CI: 0.03, 2.57) is considered positively and significantly associated with higher HSBS scores (Table 4). While the age of the bite victim had a significant p-value (0.0001), this association is not considered to be significant since the confidence interval crosses zero.

**Table 4:** Linear regression analysis results of factors significantly associated with differences in Healthcare-seeking Behavior Score among 85 canine-bite victims, Rabies Knowledge, Attitudes, and Practices Survey, Bangladesh, 2018.

		Bivariate Associations					Multivariate Associations ^b^			
VARIABLES	n^a^	β_1_	95% C.I. L	95% C.I. U	p-value	Adj R-Sq	β_1_	95% C.I. L	95% C.I. U	p-value
**Age of Bite Victim**	21	0.01	-0.01	0.03	0.37	0.00	0.01	-1.16	3.37	0.0001
**Anatomical Location of Bite on Victim**										
*Chest/Torso*	3 (3.5%)	1.98	0.38	3.57	**0.02**	0.06	0.48	-1.97	2.94	0.70
*Arm/Hand*	75 (88.2%)	-0.57	-1.51	0.36	0.23	0.01	*referent*			
**Region**										
*Chittagong*	15 (17.6%)	0.94	0.17	1.71	**0.02**	0.05	1.30	0.03	2.57	**0.045**
*Sreepur*	42 (49.4%)	-0.42	-1.02	0.18	0.17	0.01	*referent*			
**Distance of Travel to Nearest Location of Rabies Vax.**										
*≤5 km*	36 (42.4%)	-0.19	-0.80	0.43	0.55	-0.01	*referent*			
*6–20 km*	20 (23.5%)	0.73	0.03	1.43	0.04	**0.04**	0.62	-0.28	1.51	0.17

^a^ Count (percentage) presented for categorical variables & average presented for continuous variables; total of n = 85 canine-bite victims reported within a year prior to the time of survey administration

^b^ Adjusted R-Square = 0.0206; p-value cutoff ≤ 0.05

β values reflect the difference in health seeking behavior score (HSBS)among those with and without the independent variable of interest, controlling for other factors. HSBS ranges from 0 to 5. Bite victims are awarded one point each for: 1) washing of the wound, 2) seeking medical care, 3) initiating PEP, 4) completing PEP, and 5) receiving RIG). Bite victims were assigned half-points for missing responses

### 3.3 Attitudes towards post-exposure healthcare-seeking behaviors

Attitudes about specific healthcare-seeking behaviors differed significantly based on the respondent’s familiarity with the dog (Table 5). In hypothetical scenarios of a dog bite, respondents who were unable to recognize the dog were 1.2 times as likely to seek rabies PEP (p-value <0.0001), 1.1 times as likely to wash their wound (p-value 0.0019), 1.4 times as likely to call a veterinarian (p-value <0.0001), 1.3 times as likely to isolate the dog for observation (p-value <0.0001), and 1.6 times as likely to submit the animal for testing (p-value 0.0023) than respondents who recognized the hypothetical biting dog.

**Table 5:** Attitudes towards post-exposure healthcare-seeking behaviors among respondents from non-bite reporting households, Rabies Knowledge, Attitudes, and Practices Survey, Bangladesh, 2018.

What would you do if you were bitten by a dog that you DO NOT recognize or own? ^a^	n ^b^	%	What would you do if you were bitten by a dog that you recognize or own? ^a^	n ^b^	%	Risk Ratio	p-value ^c^
Wash the wound	1427	60.2%	Wash the wound	1266	53.4%	1.1	0.0019
Consult with a traditional healer	410	17.3%	Consult with a traditional healer	456	19.2%	0.9	0.1182
Call a veterinarian	728	30.7%	Call a veterinarian	513	21.6%	1.4	<0.0001
Call a medical doctor	1179	49.7%	Call a medical doctor	1094	46.2%	1.1	0.0746
Receive rabies PEP	945	39.9%	Receive rabies PEP	762	32.2%	1.2	<0.0001
Isolate the animal for observation	785	33.1%	Isolate the animal for observation	599	25.3%	1.3	<0.0001
Submit the animal for rabies testing	106	4.5%	Submit the animal for rabies testing	66	2.8%	1.6	0.0023
Kill the animal	32	1.4%	Kill the animal	34	1.4%	1.0	0.8072
Nothing	20	0.8%	Nothing	25	1.1%	0.8	0.4614

^a^ Multiple responses were allowed, column totals may not add up to 100

^b^ Total of n = 2370 households reporting

^c^ P-values resulting from chi -square analysis

### 3.4 Willingness to pay for rabies PEP

**Fig 5:**
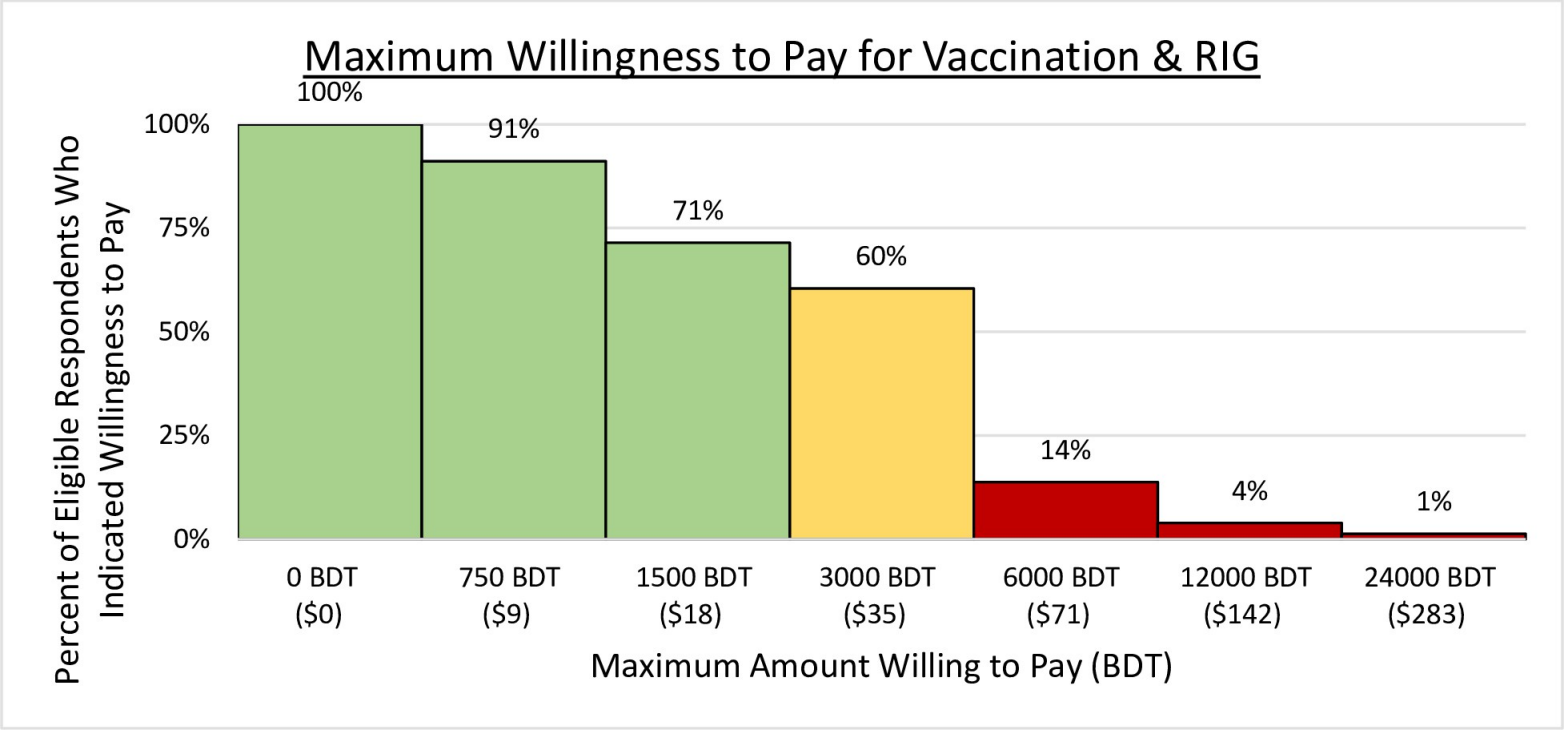
Maximum willing to pay for rabies vaccine and RIG; RIG = Rabies immunoglobulin protein.

The mean amount that respondents were willing to pay for a 4-dose course of rabies vaccine after an exposure is equivalent to approximately $25.65 USD (2,201.34 BDT). The average amount that respondents were willing to pay for RIG is equivalent to approximately $24.66 USD (2,116.38 BDT). The combined average that respondents were willing to pay for both vaccine and RIG was approximately $50.32 USD (4,318.58 BDT). The combined cost of both rabies vaccine and RIG would need to be less than $18 USD (1,544.80 BDT) in order for 70% of the study population to be willing to pay for PEP (Fig 5).

Linear regression modelling was used to determine if there was an association between the maximum amount respondents were willing to pay for rabies vaccine and RIG and the knowledge and wealth scores of the respondents. Using t-tests, there was no significant association between the maximum amount respondents were willing to pay and rabies knowledge (p-value = 0.91) nor household wealth score (p-value 0.94).

## 4. Discussion

Bangladesh continues to report one of the highest rates of human rabies in all of Asia, despite providing approximately 250,000 post-exposure prophylaxis (PEP) regimens to bite victims annually [9]. In an effort to understand barriers to rabies PEP and causes of the continued high mortality, a KAP survey (n = 2,447) was conducted in four urban and peri-urban communities in Dhaka and Chittagong divisions, representing over 13,000 residents. We found an estimated dog bite rate that ranged from 456.9 bites per 100,000 people to 1096.6 bites per 100,000 people by district, reflecting a comparable bite rate to other Asian counties such as India and Nepal, where bite rates are estimated to be 692.6 per 100,000 people and 335 per 100,000 people, respectively [3].

We found a higher dog bite rate among this urban and peri-urban study population compared to previous publications from Bangladesh [11]. Bite rates reported in our study are 2 to 10 times as high as those published in a 2012 study from only rural Bangladesh communities by Hossein et al [14]. None of the communities interviewed during the 2012 study were visited in this 2018 study. Urban/rural differences in dog ownership and bite rates have been noted in other countries, and the differences noted here reflect similar findings. Furthermore, this study shows higher frequencies of wound washing (46% vs 2.4%) and vaccine initiation (56% vs 28.5%) among bite victims compared to what has been seen in a previous study of rural Bangladesh [11]. Urban and rural settings, therefore, may have different rabies exposure characteristics and country-level strategies for rabies elimination (e.g. dog vaccination campaigns or PEP allocation) should seek to take into consideration these heterogeneities in dog bites and rabies exposures. Given differences across the urban-peri-urban gradient, as well as differences between districts sampled in this study, one set of parameter values for estimating human rabies burden applied to the entire country may not be accurate.

### 4.1 Strategies for bite prevention

Rabies knowledge among respondents was lower than expected, given the relatively high education level and widespread rabies endemicity throughout the country. Key concepts of rabies control that were lacking among respondents included awareness of the risk of rabies and proper wound washing after a bite. These key concepts should be incorporated into further comprehensive rabies prevention programs.

The majority of bite victims were children under the age of 14. Of the 37 bites reported in children 0–14 years, all but two of the children were bitten by dogs that did not belong to their family. The majority (73%) of bites came from known, community dogs, followed by unrecognized dogs (19%) and a dog owned by a neighbor (3%). Additional studies in Bangladesh on causes of childhood bites from unowned dogs are warranted as well as the evaluation of preventive measures to reduce rabies exposures and deaths among children. For instance, immediate preventative actions could include targeted education of children focused on appropriate interactions with dogs, particularly dogs that children do not recognize or are not owned by their families, in order to prevent bites [24].

Over 80% of dog bite victims were bitten by a free-roaming dog. This finding is consistent with other reports from Bangladesh that have found a large free-roaming dog population in relation to a relatively low dog-ownership rate [7, 11]. While improved bite-prevention education is clearly warranted in Dhaka and Chittagong, efforts to manage the free-roaming dog population should also be considered. Dog population management is complex and must include consideration for the humane removal of unhealthy animals from the population. However, there are very few examples where sterilization programs have resulted in sustained reductions in the dog population [25–27]. Similar to the evolution of the current humane dog population management practices in the United States, Bangladesh should consider engaging with local animal welfare organizations to develop practices and policies that result in humane dog population management while adequately addressing the public health risks inherent in free-roaming dog populations [28].

### 4.2 Post-bite healthcare-seeking behaviors and deaths following bites

Post-bite healthcare-seeking behaviors and PEP completion rates among bite victims were much higher than other reports from Asia, where 50% sought advice from a medical doctor, PEP was completed in 85% of those receiving intradermal rabies vaccination, and 22% received RIG [10, 29]. This may in part be due to the large network of PEP centers in Bangladesh, as well as the fact that PEP is provided nearly free-of-cost by the government [9].

We identified a large proportion of the bite victim sub-population (60%) with high-risk bites that did not complete PEP, even though they sought medical care. One bite victim did not initiate PEP despite being bitten in the arm by their family dog who died within ten days of the bite. When asked why this person did not receive PEP, the respondent stated that no PEP was available at the facility. Although this individual was still alive at the time of the survey 2 months after the bite, we were unable to follow up with this induvial as identifying information was not collected. Findings from this KAP survey should inform additional studies to characterize high-risk populations and the barriers they experience in seeking appropriate healthcare and rabies PEP.

This survey identified three individuals who were bitten and died or were thought to have been dead at the time of the survey, and were between the ages of 1 and 12. Both the 12-year-old and the 1-year-old bite victims are consistent with the probable rabies case definition provided by the WHO which is a suspected case plus a reliable history of contact [1]. Assuming that these two deaths were attributed to rabies, the study area had a 1-year rabies death rate of 14.8 per 100,000 persons (95% Confidence Interval 2.5–48.8). This figure is consistent with previous estimates and supports existing data that Bangladesh has one of the highest rates of human rabies deaths in the world [3].

### 4.3 A Risk-based approach to post-exposure prophylaxis

As of 2017, WHO recommends a risk-based approach when deciding to administer rabies PEP to more efficiently utilize PEP stockpiles, particularly when costs or supply of PEP may lead to poor access for those that truly need it [1]. However, Bangladesh currently lacks national guidelines for a risk-based approach, instead recommending provision of PEP to all bite victims that are treated at bite centers. Practical experience has shown, and been supported with data presented here, that nearly everyone who attends a DRPCC clinic is provided intradermal PEP, if it is available. However, the sustainability of this massive distribution of PEP is questionably dependent upon government-supported budgets and prone to regional and national supply shortages as was the case for at least one bite victim in this study. Examples of large-scale implementation of these risk-based (i.e. integrated bite case management) systems are rare for low- and middle-income countries [30]. Findings from this survey support that a risk-based approach could be beneficial to triage bite victims based on risk-level and improved post-bite observation of dogs is likely feasible and could result in substantial PEP cost-savings of at least $1.4 million USD (1.2 billion BDT). This approach could also be helpful in reducing the burden on health care facilities. According to The World Bank, Bangladesh has 0.5 physicians per 1,000 people which is higher than what we have seen in other countries who have successfully implemented a risk based approach [13].

The findings from this study show great promise for the potential to implement risk-based PEP approaches, such as integrated bite case management (IBCM) programs. Other programs that have instituted these risk-based approaches have shown similar capacity to follow-up and assess biting dogs [30,31]. Despite a lack of any formal national guidance for the assessment of biting animals, bite victims are able to identify the health status of the biting dog. A study of IBCM implementation in a low-income country found that the complete cost of case investigation (staff, supplies, quarantine, testing) was $24 USD (2,059.74 BDT) per animal assessed [13]. If the WHO-recommended risk-based approach were implemented, and PEP could be safely avoided for persons assessed to have no risk for rabies, the resulting cost-savings could support as many as 56,568 IBCM animal case investigations each year [13]. Initial investment in high-quality surveillance and response programs can be daunting, but continued administration of high-volumes of PEP to persons with no clear risk of rabies poses an undue burden on the government, particularly when these funds could support additional critical rabies control infrastructure development.

There is some evidence to support that bite victims in Bangladesh can afford to contribute towards the costs for rabies PEP. However, any implementation of patient-costs for PEP should be very carefully evaluated. Policies that could negatively impact PEP access for persons with legitimate rabies exposures should be avoided. Fees based on level of risk and ability to pay should be considered [32]. When patients are assessed to have had a true rabies virus exposure, it is the responsibility of public health authorities to ensure that they have access to appropriate treatment.

As with similar KAP studies, this study has its limitations. Only one person from each surveyed household was interviewed who may or may not be the primary decision maker. As such their attitudes and knowledge of rabies may not be reflective of that of the whole household. This person also provided information regarding all bites that had occurred in the household in the year prior to the survey and therefore may be subject to differential recall bias. For instance, participants may be more likely to recall severe bites or bites from an unknown dog rather than mild bites or bites from their owned dog. Confirmation biases may also be contributing to improved health seeking behaviors.

## 5. Conclusion

Findings presented here provide useful information regarding bite occurrences, healthcare-seeking behaviors, and a need for strategies to increase rabies awareness. This study confirmed a high human rabies death rate in Bangladesh despite a high rate of PEP access and completion rates. While PEP is widely offered throughout the country, results presented here show that the country is not immune to errors in distribution. Implementation of a risk-based approach with an educational component would very likely lead to more efficient PEP utilization, and the cost-savings could be diverted to support advancements in rabies surveillance through development of a program such as integrated bite case management [30].

## Supporting information

S1 Text Knowledge, Attitude, and Practices questionnaire. (DOCX)

S1 Table Full linear regression analysis of factors associated with differences in Healthcare-seeking Behavior Score among 85 canine-bite victims. (DOCX)

S1 Data Dataset. (XLSX)
