# Supplementary material for: Rabies healthcare-seeking behaviors of urban and peri-urban residents: Results from a rabies knowledge, attitudes, and practices survey, Bangladesh, 2018
Source: PLoS Negl Trop Dis. 2022 Aug 9;16(8):e0010634. doi: 10.1371/journal.pntd.0010634 (PMC9390912; doi:10.1371/journal.pntd.0010634)
Supplement: S1 Text — (DOCX) [file pntd.0010634.s001.docx]

**Mass rabies dog vaccination and evaluation**

**Knowledge, attitudes, and practices survey**

June 2018

Bangladesh

| **Introduction** | |
| --- | --- |
| **To the interviewee:** “Thank you for being willing to participate in this survey. I am going to start by asking you basic questions about yourself to get to know you better. Your responses will help us better understand people’s knowledge of rabies in Bangladesh. It will also help us identify barriers that make it difficult for people to seek medical care after a potential rabies exposure or proactively vaccinate their dogs to prevent exposures. Please note that your name and any other identifying information will not be collected during this survey. Participation is entirely voluntary and you may tell us to stop the survey at any time for any reason. [provide consent leaflet] | |
| 1. Indicate response below:  - Consent given - Consent not given - Adult respondent not home/available - Household missing - Household abandoned | 🡪Begin the interview  🡪Go to the next selected household  🡪Go to the next selected household and return at a later time  🡪Go to the next selected household  🡪Go to the next selected household |

| **I. Interviewer Information** | | |
| --- | --- | --- |
| **No.** | **Question** | **Response** |
| 101 | Name of data collector | ____________________________________ |
| 102 | Date (DD/MM/YYYY): | ___ ___/ ___ ___/ ___ ___ ___ ___ |
| 103 | Zone | ___ ___ |
| 104 | GPS | Latitude: __________________  Longitude: _________________ |

| **II. Demographics** | | |
| --- | --- | --- |
| **No.** | **Question** | **Response** |
| 201 | Is the respondent head of household? | □ Yes □ No |
| 202 | What is your age? | (#) ___________ years |
| 203 | What is your gender? | **□** Male **□** Female |
| 204 | Number of family members in household (including self)? | (#) _______________ |
| 205 | What is your religion? | **□** Islam **□** Hinduism **□** Buddhism  **□** Christian **□** No response  **□** Other: ____________________________ |
| 206 | What is your average monthly household expenditure? | **□** <5,500 BDT **□** 5,500–10,000 BDT  **□** 10,001–20,000 BDT **□** 20,001–40,000 BDT  **□** >40,000 BDT |
| 207 | What is the source of your drinking water? | **□** Piped inside **□** Tube well  **□** Dug well **□** Pond/River/Lake water  **□** Other: _____________________________ |
| 208 | What kind of toilet facility does your household have? | **□** Flush - to sewer or septic tank  **□** Flush - to open latrine  **□** Pit latrine **□** Hanging latrine  **□** No facility/bush/field  **□** Other: ____________________ |
| 209 | What is the material of the walls of your house? *(Enumerator assess)* | **□** Cane/palm/trunks **□** Dirt/mud  **□** Tin **□** Cement  **□** Bricks **□** Other: ____________________ |
| 210 | Does your household own the following items?  *Read and check all that apply* | \| Item \| Have \| Don’t have \| \| --- \| --- \| --- \| \| Television \|  \|  \| \| Electric fan \|  \|  \| \| Refrigerator \|  \|  \| \| Electricity \|  \|  \| \| Bank account \|  \|  \| |
| 211 | What is the highest level of education achieved by a member of your household? | **□** No education  **□** Some primary **□** Completed primary  **□** Some secondary **□** Completed secondary  **□** College education |

|  | | | **IV. Bite rates** | | | | | | | | | | | |  |
| --- | --- | --- | --- | --- | --- | --- | --- | --- | --- | --- | --- | --- | --- | --- | --- |
| **No.** |  | | | | **Question** | | | | | | | **Response** | | |  |
| 301 |  | | | | **In the past year**, have you or anyone in your household been bitten or scratched by an animal? | | | | | | | - Yes - No | - Skip to 401 | |  |
| 302 |  | | | | Please provide information on each bite or scratch event that has occurred among members of your household. *{Complete one line for every person bitten/scratched and Repeat for every bite event (e.g. if a person had multiple bites during the year, each bite event should be recorded separately)}* | | | | | | | | | |  |
| **a. Victim Age** | | **b. Year and Month of bite** | | **c. Type of Animal** | | **d. Animal was:**  **1. Mine**  **2. Neighbors**  **3. Unknown** | **e. Where on the body did the bite/scratch occur?** | **f. Type of exposure** | **g. Did the animal die within 10 days of biting?** | **h. Did you/they seek medical care?**  **Yes-med 🡪 303**  **No-trad 🡪 304** | **i. Did the person experience any complications/infections related to the bite wound?** | | | **j. Is this person still alive?** |  |
| **1.** | |  | | Dog  Cat  Other | | Mine  Neighbors  Unknown | Head/Neck  Chest  Arm/ Hand  Leg/ Foot | Bite  Scratch | ○ Yes  ○ No  ○ I don’t know | ○ Yes - medical  ○ Yes - traditional  ○ No○ No | ○ Yes  ○ No | | | ○ Yes  ○ No |  |
| **2.** | |  | | Dog  Cat  Other | | Mine  Neighbors  Unknown | Head/Neck  Chest  Arm/ Hand  Leg/ Foot | Bite  Scratch | ○ Yes  ○ No  ○ I don’t know | ○ Yes - medical  ○ Yes - traditional  ○ No | ○ Yes  ○ No | | | ○ Yes  ○ No |  |
| **3.** | |  | | Dog  Cat  Other | | Mine  Neighbors  Unknown | Head/Neck  Chest  Arm/ Hand  Leg/ Foot | Bite  Scratch | ○ Yes  ○ No  ○ I don’t know | ○ Yes - medical  ○ Yes - traditional  ○ No | ○ Yes  ○ No | | | ○ Yes  ○ No |  |
| **4.** | |  | | Dog  Cat  Other | | Mine  Neighbors  Unknown | Head/Neck  Chest  Arm/ Hand  Leg/ Foot | Bite  Scratch | ○ Yes  ○ No  ○ I don’t know | ○ Yes - medical  ○ Yes - traditional  ○ No | ○ Yes  ○ No | | | ○ Yes  ○ No |  |
| **5.** | |  | | Dog  Cat  Other | | Mine  Neighbors  Unknown | Head/Neck  Chest  Arm/ Hand  Leg/ Foot | Bite  Scratch | ○ Yes  ○ No  ○ I don’t know | ○ Yes - medical  ○ Yes - traditional  ○ No | ○ Yes  ○ No | | | ○ Yes  ○ No |  |
|  |  | | | |  | | | | | | | | | |  |
| **303** |  | | | | If the victims sought medical care, please fill out the table: | | | | | | | | | | |
|  | | | \| **Incident # (as assigned above)** \| **a. How many days occurred between when they were bitten on this occasion and medical care was sought?** \| **b. Did they receive rabies vaccine?** \| **c. How many times/days (including the first visit where vaccine was received) did you return to a clinic to receive a dose of rabies vaccine?** \| **d. Did the person receive rabies immuno-globulin?** \| **e. If the vaccine was not received, why was the vaccine not received?**   1. **Did not think it was necessary** 2. **Lack of nearby facilities to provide treatment** 3. **Lack of trained personnel at facilities** 4. **Lack of vaccines at facility** 5. **No means of transportation** 6. **Cost of vaccine** 7. **Would have to miss work** 8. **Other, specify** \| **f. If not all doses were received, why were not all doses received?**   1. **Did not think it was necessary** 2. **Adverse event occurred** 3. **Forgot** 4. **Dog was tested negative** 5. **Dog was observed in quarantine** 6. **Not pleased with original experience at facility** 7. **Distance to facility too far** 8. **Would have to miss work** 9. **Other, specify** \| \| --- \| --- \| --- \| --- \| --- \| --- \| --- \| \| **1.** \|  \| ○ Yes ○ No \|  \| ○ Yes ○ No \|  \|  \| \| **2.** \|  \| ○ Yes ○ No \|  \| ○ Yes ○ No \|  \|  \| \| **3.** \|  \| ○ Yes ○ No \|  \| ○ Yes ○ No \|  \|  \| \| **4.** \|  \| ○ Yes ○ No \|  \| ○ Yes ○ No \|  \|  \| \| **5.** \|  \| ○ Yes ○ No \|  \| ○ Yes ○ No \|  \|  \| | | | | | | | | | | | | |

| **304** | If the victim did not seek medical care, please fill out this table: |
| --- | --- |
| \| **Person #** \| **Why did they not seek medical care for this bite at a healthcare facility?**   1. **Did not think it was necessary** 2. **Lack of nearby facilities to provide treatment** 3. **Lack of trained personnel at facilities** 4. **Lack of vaccines at facility** 5. **No means of transportation** 6. **Cost of vaccine** 7. **Would have to miss work** 8. **Traditional healer provided sufficient care** 9. **Other, specify** \| \| --- \| --- \| \| **1.** \|  \| \| **2.** \|  \| \| **3.** \|  \| \| **4.** \|  \| \| **5.** \|  \| | |

| **IV. Attitudes: dog bites and ownership** | | | | | | | | | | |  |  |
| --- | --- | --- | --- | --- | --- | --- | --- | --- | --- | --- | --- | --- |
| **No.** | | **Question** | | | | | **Response** | | | |  |  |
| 401 | | What are good reasons to own a dog?  *Check all that apply* | | | | | **□** Companionship **□** Security **□** Hunting  **□** Herding **□** Protect livestock **□** Protect crops  **□** Breeding/Sale **□** No good reason  **□**Other: __________________________ | | | |  |  |
| *For questions 402 to 405, please state whether you agree or disagree with the following statements:* | | | | | | | | | | | |  |
| 402 | | | | People who don’t vaccinate their dogs against rabies are irresponsible owners | | **□** Agree **□** Disagree **□** No opinion | | | | | |  |
| 403 | | | | Dogs are unclean. | | **□** Agree **□** Disagree **□** No opinion | | | | | |  |
| 404 | | | | I am afraid of dogs. | | **□** Agree **□** Disagree **□** No opinion | | | | | |  |
| 405 | | | | I am afraid of getting rabies from dogs. | | **□** Agree **□** Disagree **□** No opinion | | | | | |  |
|  | | | |  | |  | | | | | |  |
| 406a | | What would you do if you were bitten by a dog that you **recognize or own**?  *Don’t read choices. Mark all that apply* | | | | | **□** Wash wound **□** Consult with a traditional healer  **□** Consult with a village health care worker  **□** Consult with an animal health worker  **□**Contact police **□** Seek care at medical facility  **□**Seek rabies post exposure prophylaxis  **□** Isolate the dog for observation  **□** Kill the dog  **□** Nothing  **□** Other, describe: ________________  **□** Decline to answer <skip to 407a> | | | |  |  |
| 406b | | What would you do if you were bitten by a dog that you **recognize or own**?  *Now read choices. Mark all that apply* | | | | | Would you….  **□** Wash wound **□** Consult with a traditional healer  **□** Consult with a village health care worker  **□** Consult with an animal health worker  **□**Contact police **□** Seek care at medical facility  **□**Seek rabies post exposure prophylaxis  **□** Isolate the dog for observation  **□** Kill the dog  **□** Nothing  **□** Other, describe: ________________ | | | |  |  |
| 407a | | What would you do if you were bitten by a dog that you **do not** recognize or own?  *Don’t read choices. Mark all that apply* | | | | | **□** Wash wound **□** Consult with a traditional healer  **□** Consult with a village health care worker  **□** Consult with an animal health worker  **□**Contact police **□** Seek care at medical facility  **□**Seek rabies post exposure prophylaxis  **□** Isolate the dog for observation  **□** Kill the dog  **□** Nothing  **□** Other, describe: ________________  **□**Decline to answer <skip to 408> | | | |  |  |
| 407b | | What would you do if you were bitten by a dog that you **do not** recognize or own?  *Now read choices. Mark all that apply* | | | | | Would you….  **□** Wash wound **□** Consult with a traditional healer  **□** Consult with a village health care worker  **□** Consult with an animal health worker  **□**Contact police **□** Seek care at medical facility  **□**Seek rabies post exposure prophylaxis  **□** Isolate the dog for observation  **□** Kill the dog  **□** Nothing | | | |  |  |
| 408 | | If you saw a dog in your village that looked sick, what would you do?  *Read and mark all that apply* | | | | | **□** Consult with a village health care worker  **□** Consult with an animal health worker  **□** Contact a friend **□** Avoid the animal  **□** Scare/ shoo the dog away **□** Kill the dog  **□** Kill and eat the dog **□**Submit animal for testing  **□** Nothing **□** Decline to answer  **□** Other, describe: ________________ | | | |  |  |
| 409 | | What type of exposure would prompt you to get medical treatment at a health care facility?  *Read and mark all that apply* | | | | | **□** Unprovoked scratch from owned dog or cat  **□** Unprovoked bite from owned dog or cat  **□** Provoked scratch from stray dog or cat  **□** Unprovoked bite from stray dog or cat  **□** Don’t know  **□** Other: ____________________________ | | | |  |  |
| **Attitude: Willingness to pay** | | | | | | | | | | |  |  |
| 410 | | You have received a severe bite on your leg from a dog. Because of this, your healthcare provider recommended you to get post-exposure prophylaxis. This will consist of receiving 4 doses of vaccine and plus one initial dose of rabies immunoglobulin. Because it is a severe exposure, vaccine alone may not provide equivalent protection against rabies. Only the combination of vaccine and immune globulin can guarantee you won’t get rabies.  Would you be willing to pay 2,500 BDT for a full course (4 doses) of the rabies vaccine? | | | | | | | | |  |  |
|  | | - Yes | | | | | - No | | | |  |  |
|  | | A.1 If *Yes,* Could you pay 3,000 BDT? | | | | | B.1 If *No, Could you* pay 2,000 BDT? | | | |  |  |
|  | | **□** Yes **□** No | | | - Skip to 411 | | **□** Yes **□** No | | - Skip to 411 | |  |  |
|  | | A.2 If *Yes,* Could you pay 3,500 BDT? | | | | | B.2 If *No,* Could you pay 1,500 BDT? | | | |  |  |
|  | | **□** Yes **□** No | | | - Skip to 411 | | **□**Yes **□** No | | - Skip to 411 | |  |  |
|  | | A.3 If *Yes,* If you are willing and able to pay more than 3,500 BDT, please specify the amount you are willing to pay: _____________ BDT | | | | | B.3 If you are unable or unwilling to pay 1,500 BDT, please specify the amount you are willing to pay:  ______________ BDT | | | |  |  |
| 411 | | In addition to paying [piped from previous question] for the vaccine, are you willing and able to pay 3,500 BDT for immunoglobulin? | | | | | | | | |  |  |
|  | | - Yes | | | | | - No | | | |  |  |
|  | | A.1 If *Yes,* Could you pay 4,500 BDT? | | | | | B.1 If *No, Could you* pay 2,500 BDT? | | | |  |  |
|  | | **□** Yes **□** No | | | - Skip to 412 | | **□** Yes **□** No | | - Skip to 412 | |  |  |
|  | | A.2 If *Yes,* Could you pay 5,500 BDT? | | | | | B.2 If *No,* Could you pay 1,500 BDT? | | | |  |  |
|  | | **□** Yes **□** No | | | - Skip to 412 | | **□** Yes **□** No | | - Skip to 412 | |  |  |
|  | | A.3 If *Yes,* Could you pay 6,500 BDT? | | | | | B.4 If *No,* Could you pay 500 BDT? | | | |  |  |
|  | | **□** Yes **□** No | | | - Skip to 412 | | **□** Yes **□** No | | - Skip to 412 | |  |  |
|  | | A.5 If *Yes,* If you are willing and able to pay more than 6,500 BDT, please specify the amount you are willing to pay: _____________ BDT | | | | | B.5 If you are unable and unwilling to pay 500 BDT, please specify the amount you are willing to pay:  ______________ BDT | | | |  |  |
| **V. Knowledge: Healthcare access** | | | | | | | | | | | | |
| **No.** | | | **Question** | | | | | **Response** | | | | |
| 501 | | | How far do you travel for routine medical care? | | | | | **□** <1 km **□** 1-5 km **□** 6-10 km  **□** 11-20 km **□** 21-30 km **□** > 31 km  **□** I don’t know  **□** Declined to answer | | | | |
| 502 | | | What is the primary mode of transportation you would use to go to a health facility? | | | | | **□** Walk **□** Rickshaw **□** CNG  **□** Bus **□** Personal vehicle  **□** Other_______________ | | | | |
| 503 | | | Do you know where you can receive medical treatment for rabies exposures (i.e. animal bite)? | | | | | **□** Yes  **□** No **□** Don’t know | | - Skip to 506 | | |
| 504 | | | What facility would you go to? | | | | | **□** District Hospital **□** Infectious Disease Hospital  **□** Other: ___________________________- | | | | |
| 505 | | | How far do you need to travel to receive medical treatment for a rabies exposure? | | | | | **□** <1 km **□** 1-5 km **□** 6-10 km  **□** 11-20 km **□** 21-30 km **□** > 31 km  **□** I don’t know  **□** Declined to answer | | | | |
| 506 | | | How much would it cost to travel to receive medical treatment for a rabies exposure? | | | | | ______________ BDT  **□** I don’t know | | | | |
| 507a | | | What are the primary obstacles for getting medical treatment for rabies exposures in your community?  *Do not read choices. Mark all that apply* | | | | | **□** Lack of facilities to provide treatment  **□** Lack of trained personnel at facilities  **□** Lack of medicines at facilities  **□** Prefer traditional healers  **□** No means of transportation  **□** No money to pay for treatment  **□** Can’t miss work  **□** I don’t know **□** Other:___________________ | | | | |
| 507b | | | What are the primary obstacles for getting medical treatment for rabies exposures in your community?  *Now read choices. Mark all that apply* | | | | | **□** Lack of facilities to provide treatment  **□** Lack of trained personnel at facilities  **□** Lack of medicines at facilities  **□** No means of transportation  **□** No money to pay for treatment  **□**Can’t miss work | | | | |
| **Knowledge: Rabies and rabies vaccination** | | | | | | | | | | | | |
| 508 | | | Have you ever heard of a disease called ‘rabies’? | | | | | **□** Yes  **□** No **□** Don’t know | | - Skip to 601 | | |
| 509 | | | Do you know anyone in your community who has ever died from a disease called ‘rabies’? | | | | | **□** Yes  **□** No **□** Don’t know | | | | |
| 510 | | | How severe is the disease called ‘rabies’? | | | | | **□** Mild **□** Somewhat severe  **□** Very severe, but recoverable  **□** Very severe, fatal  **□** Don’t know **□** Decline to answer | | | | |
| 511 | | | Of the following, which disease do you most fear getting: *Select one* | | | | | **□** Malaria **□** HIV/AIDs **□** Rabies  **□** Tuberculosis **□** Influenza **□** Don’t know  **□** Other:_____________ | | | | |
| 512 | | | How do humans get rabies from an infected animal?  *Read choices. Mark all that apply* | | | | | **□** Bite **□** Scratch **□** Observing the animal  **□** Touching the animal **□** Contact with blood  **□** Contact with saliva **□** Contact with urine/feces  **□** Don’t know **□** Decline to answer  **□** Other: _______________________________ | | | | |
| 513 | | | On a scale of 1 to 5, with 1 being little to no risk of rabies from that animal, list the rabies risk of each animal | | | | | **□** Dogs:  **□** Cats:  **□** Livestock:  **□** Bats:  **□** Rodents | | 1 2 3 4 5  1 2 3 4 5  1 2 3 4 5  1 2 3 4 5  1 2 3 4 5 | | |
| 514 | Do you think it is important that a dog be vaccinated against rabies? | | | | | | | **□** Yes **□** No -> Skip to 516 | | | | |
| 515 | How often should a dog be vaccinated against rabies? | | | | | | | **□** At least once a year **□** Every other years  **□** 2-3 times per lifetime **□** Once in a lifetime | | | | |
| 516 | Do you think the rabies vaccine poses any risks to a dog? | | | | | | | **□** Yes **□** No -> Skip to 601 | | | | |
| 517 | What are the risks to dogs from being vaccinated against rabies? | | | | | | | **□** Produces anxiety in the dog  **□** Produces bleeding in the dog  **□** Can cause rabies  **□** Can cause other infectious diseases  **□** Can cause diarrhea/vomiting  **□** Can cause other side effects  **□** Death **□** I don’t know **□** No response  **□**Other: _____________________________ | | | | |

| **VI. Dog ownership** | | |
| --- | --- | --- |
| **No.** | **Question** | **Response** |
| 601 | Does your household currently own any dogs? | **□** Yes **□** No -> Skip to 609 |
| 602 | How many dogs live in your household currently? | ____________________ dogs |
| 603 | What level of care does your household provide for your dogs?  *Select all that apply* | **□** None **□** Food **□** Water  **□** Shelter **□** Veterinary care  **□** No response  **□** Other: _____________________ |

| 604 | Please provide all the information for all the dogs that live in your household, including their vaccination status: |
| --- | --- |
| \| **Dog** \| **a. Age** \| **b. Sex**  **-M**  **-F** \| **c. Type of dog:**   1. **Purebred** 2. **Mixed** \| **d. Confinement status:**   1. **Always on property** 2. **Roaming, sometimes** 3. **Roaming, always** \| **e. What is the primary reason you own this dog?**   1. **Security (guard dog)** 2. **Companionship** 3. **Hunting** 4. **Herding** 5. **Protect crops and livestock from other animals** 6. **Ward off spirits** 7. **Wealth asset** 8. **Other, describe** \| **f. # rabies vaccinations during its lifetime** \| **g. Was this dog vaccinated against rabies in the past year?** \| **h. For the most recent rabies vaccine this dog received, where did you get the vaccine from?**   1. **At vet facility** 2. **From vaccination campaign** 3. **Other, describe** \| **i. Was this dog vaccinated in the recent campaigns?** \| **k. Did this dog come home at any point in the last week with a paint mark on him/her?** \| **l. If dog has not been vaccinated why not?**  **1.It is not important to vaccinate this dog**  **2.This dog is too old**  **3.This dog is too young**  **4.This dog was sick**  **5.This dog was not home at the time**  **6.This dog would get into fights with other dogs**  **7.This dog is too difficult to transport**  **8.Other, specify** \| \| --- \| --- \| --- \| --- \| --- \| --- \| --- \| --- \| --- \| --- \| --- \| --- \| \| 1. \|  \|  \|  \|  \|  \|  \| ○ Yes  ○ No \|  \| ○ Yes  ○ No  ○ Don’t know \| ○ Yes  ○ No  ○ Don’t know \|  \| \| 2. \|  \|  \|  \|  \|  \|  \| ○ Yes  ○ No \|  \| ○ Yes  ○ No  ○ Don’t know \| ○ Yes  ○ No  ○ Don’t know \|  \| \| 3. \|  \|  \|  \|  \|  \|  \| ○ Yes  ○ No \|  \| ○ Yes  ○ No  ○ Don’t know \| ○ Yes  ○ No  ○ Don’t know \|  \| \| 4. \|  \|  \|  \|  \|  \|  \| ○ Yes  ○ No \|  \| ○ Yes  ○ No  ○ Don’t know \| ○ Yes  ○ No  ○ Don’t know \|  \| \| 5. \|  \|  \|  \|  \|  \|  \| ○ Yes  ○ No \|  \| ○ Yes  ○ No  ○ Don’t know \| ○ Yes  ○ No  ○ Don’t know \|  \| \| 6. \|  \|  \|  \|  \|  \|  \| ○ Yes  ○ No \|  \| ○ Yes  ○ No  ○ Don’t know \| ○ Yes  ○ No  ○ Don’t know \|  \| | |

| *For questions 605 to 609, please state whether you agree or disagree with the following statements (DOG OWNERS ONLY):* | | | | |
| --- | --- | --- | --- | --- |
| 605 | My dogs are a part of my family. | | **□** Agree **□** Disagree **□** No opinion | |
| 606 | If my dog were to die, it would be easy to replace him/her. | | **□** Agree **□** Disagree **□** No opinion | |
| 607 | I feel affection for my dogs. | | **□** Agree **□** Disagree **□** No opinion | |
| 608 | My dog is accustomed to walking on a leash. | | **□** Agree **□** Disagree **□** No opinion | |
| 609 | Other people (family, friends, veterinarians) have encouraged me to vaccinate my dogs | | **□** Agree **□** Disagree **□** No opinion | |
|  |  | |  | |
| 610 | | Did your household own any dogs that died **in the past year?** | | **□** Yes **□** No -> Skip to 613 |
| 611 | | How many dogs from your household died in the past year? | | ____________________ dogs |
| 612 | | *For dogs who died,* fill out the following table: | | |
| \| **Dog** \| **Month of death** \| **Cause of death**   1. **Hit by car** 2. **Poisoned** 3. **Rabies** 4. **Other disease/illness** 5. **Age related** 6. **I don’t know** 7. **Other, specify** \| **In the past year,** did any of those dogs **die** shortly after displaying any of the following symptoms? \| \| \| \| \| \| --- \| --- \| --- \| --- \| --- \| --- \| --- \| --- \| \| **Hypersalivation** \| **Aggression** \| **Biting (people or animals)** \| **Difficulty walking** \| **Change in bark** \| \| 1 \|  \|  \| ○ Yes ○ No \| ○ Yes ○ No \| ○ Yes ○ No \| ○ Yes ○ No \| ○ Yes ○ No \| \| 2 \|  \|  \| ○ Yes ○ No \| ○ Yes ○ No \| ○ Yes ○ No \| ○ Yes ○ No \| ○ Yes ○ No \| \| 3 \|  \|  \| ○ Yes ○ No \| ○ Yes ○ No \| ○ Yes ○ No \| ○ Yes ○ No \| ○ Yes ○ No \| \| 4 \|  \|  \| ○ Yes ○ No \| ○ Yes ○ No \| ○ Yes ○ No \| ○ Yes ○ No \| ○ Yes ○ No \| \| 5 \|  \|  \| ○ Yes ○ No \| ○ Yes ○ No \| ○ Yes ○ No \| ○ Yes ○ No \| ○ Yes ○ No \| \| 6 \|  \|  \| ○ Yes ○ No \| ○ Yes ○ No \| ○ Yes ○ No \| ○ Yes ○ No \| ○ Yes ○ No \| \| 7 \|  \|  \| ○ Yes ○ No \| ○ Yes ○ No \| ○ Yes ○ No \| ○ Yes ○ No \| ○ Yes ○ No \| | | | | |
| 613 | | Do you provide care for any dogs that you do **NOT** own?  *Mark all that apply* | | **□** No **□** Food **□** Water **□** Shelter  **□** Veterinary care  **□** Other: ___________________________  **□** No response |

| **VII. Campaign Awareness and Barriers to dog rabies vaccination** | | | | | | |
| --- | --- | --- | --- | --- | --- | --- |
| **No** | | **Question** | | **Response** | | |
| 701 | | Did you know that a dog rabies vaccination campaign was taking place on (*date*)? | **□** Yes **□** No -> Skip to 704 | | |  |
| 702 | | Did you hear about the campaign before, during, or after the campaign took place? | **□** Before **□** During  **□** After -> Skip to 704 or end | | |  |
| 703 | | If before, how did you hear about the campaign?  *Read off and select all that apply* | \|  \| Before \| After \| \| --- \| --- \| --- \| \| Print media (newspapers, posters, leaflets) \|  \|  \| \| Mikeing \|  \|  \| \| Radio \|  \|  \| \| Friend/neighbor \|  \|  \| \| Health care worker \|  \|  \| \| Other, specify____________ \|  \|  \| | | |  |
| End of survey for all households who do not own dogs  ***(‘No’ to 601 AND 612)*** | | | | | | |
| 704 | | Would you prefer to have your dog vaccinated during a campaign or at the vet’s office?  **□** No preference 🡪 Skip to 707  **□** Would not vaccinate 🡪 Skip to 707 | | | | |
|  | | **□** Campaign  **□** Veterinarian office | |  | | |
| 705 | | Do you think there is a difference in vaccine quality between the vaccines at the campaign and vaccines from the vet’s office? If yes, which is better? | | **□** Yes – vet is better  **□** Yes – campaign is better  **□** No – they are the same quality  **□** Don’t know | | |
| 706 | | Do you think there is a difference in staff training between people in campaigns and people at the vet’s office? If yes, which are better trained? | | **□** Yes – vet is better  **□** Yes – campaign is better  **□** No – they are the same quality  **□** Don’t know | | |
| 707 | | If rabies vaccine were offered to your dog, would you or someone from your household be willing and able to travel 2 km with your dog to a vaccination clinic? | | | | |
|  | **□**Yes **□**No | | | |  |  |
|  | A.1 If *Yes,* Would you travel 3 km? | | | B.1 If *No,* Would you travel 1.5 km? |  |  |
|  | **□** Yes **□** No -> Skip to 711 | | | **□** Yes **□** No -> Skip to 711 |  |  |
|  | A.2 If *Yes,* Would you travel 5 km? | | | B.2 If *No,* Would you travel 1 km? |  |  |
|  | **□** Yes **□** No -> Skip to 711 | | | **□** Yes **□** No -> Skip to 711 |  |  |
|  | A.3 If *Yes,* Would you travel 7 km? | | | B.3 If *No,* Would travel 800 meters? |  |  |
|  | **□** Yes **□** No -> Skip to 711 | | | **□** Yes **□** No -> Skip to 711 |  |  |
|  | A.3 If *Yes,* Would you travel 10 km? | | | B.4 If *No,* Would you travel 500 m? |  |  |
|  | **□** Yes **□** No -> Skip to 711 | | | **□** Yes **□** No -> Skip to 711 |  |  |
|  | A.5 If *Yes,* If you are willing and able to pay more than 10 km, please specify the amount you are willing to travel:  _____________ km | | | B.5 If you are unable or unwilling to travel 500 m, please specify the amount you are willing to travel:  ______________ m |  |  |
| ***If the campaign was a mobile central point:*** | | | | | | |
| 708 | | Did you or someone from your household go to the campaign? | | **□** Yes **□** No -> skip to 712 | | |
| 709 | | How far was the campaign from your house? | | _____________ km | | |
| 710 | | How long did you/your household member have to wait in line at the campaign? | | _____________ min | | |
| 711 | | Was your dog bitten while standing in line at the campaign? | | **□** Yes **□** No | | |
| 712 | | *If no to 708,* Why did you not go to the campaign?  *Do not read choices. Select all that apply* | | **□** I did not know about it  **□** It is not important to vaccinate against rabies  **□** I was unavailable or had to work at the time of the campaign  **□** I could not find my dogs  **□** The campaign was far from my house  **□** I saw long lines at the campaign  **□** Transporting my dogs to the campaign is difficult  **□** I do not trust the vaccine  **□** I did not trust the healthcare workers  **□** I did not want to pay for the vaccine  **□** There is risk of infection from unclean needles  **□** My dogs have already received a vaccine  **□** There is a risk of dog fight at the campaign  **□** It is unnecessary to spend extra time on dogs  **□** Other: _______________________________ | | |
| End of survey. Thank you for your time and participation!!! | | | | | | |
