# Supplementary material for: Rabies healthcare-seeking behaviors of urban and peri-urban residents: Results from a rabies knowledge, attitudes, and practices survey, Bangladesh, 2018
Source: PLoS Negl Trop Dis. 2022 Aug 9;16(8):e0010634. doi: 10.1371/journal.pntd.0010634 (PMC9390912; doi:10.1371/journal.pntd.0010634)
Supplement: S1 Table — (DOCX) [file pntd.0010634.s002.docx]

**S1 Table**

Linear regression analysis of factors associated with differences in Healthcare-seeking Behavior Score among 85 canine-bite victims, Rabies Knowledge, Attitudes, and Practices Survey, Bangladesh, 2018

|  |  | **Bivariate Associations** | | | | | **Multivariate Associations ^b^** | | | |
| --- | --- | --- | --- | --- | --- | --- | --- | --- | --- | --- |
| **VARIABLES** | **n^a^** | **β_1_** | **95% C.I. L** | **95% C.I. U** | **p-value** | **Adj R-Sq** | **β_1_** | **95% C.I. L** | **95% C.I. U** | **p-value** |
| **Age of Bite Victim** | 21 | 0.01 | -0.01 | 0.03 | 0.37 | 0.00 | 0.01 | -1.16 | 3.37 | 0.0001 |
| **Cost of Travel to Nearest Location of Rabies Vax.** | 1621 | 0.00 | 0.00 | 0.00 | 0.09 | 0.02 | 0.00 | 0.00 | 0.00 | 0.62 |
| **Biting Dog Ownership Status** |  | | | | | | | | | |
| *Owned by Bite Victim* | 4 (4.7%) | 0.16 | -1.28 | 1.60 | 0.82 | -0.01 | 0.35 | -1.33 | 2.03 | 0.68 |
| *Owned by Neighbor of Bite Victim* | 8 (9.4%) | -0.93 | -1.96 | 0.09 | 0.07 | 0.03 | -0.66 | -1.85 | 0.52 | 0.27 |
| *Unrecognized Dog* | 21 (24.7%) | 0.35 | -0.35 | 1.05 | 0.33 | 0.00 | 0.91 | -0.08 | 1.90 | 0.07 |
| *Known community dog* | 52 (61.2%) | 0.03 | -0.60 | 0.66 | 0.92 | -0.01 | *referent* |  | | |
| **Status of Biting Dog 10 Days Post-Exposure** |  | | | | | | | | | |
| *Known to Have Passed Quarantine* | 26 (30.6%) | -0.27 | -0.93 | 0.38 | 0.41 | 0.00 | *referent* |  | | |
| *Did Not Pass Quarantine* | 9 (10.6%) | 0.27 | -0.72 | 1.26 | 0.59 | -0.01 | -0.02 | -1.22 | 1.17 | 0.97 |
| *Unknown Outcome* | 50 (58.8%) | 0.14 | -0.48 | 0.75 | 0.66 | -0.01 | -0.12 | -0.92 | 0.68 | 0.77 |
| **Anatomical Location of Bite on Victim** |  | | | | | | | | | |
| *Head/Neck* | 4 (4.7%) | 0.95 | -0.47 | 2.38 | 0.19 | 0.01 | 0.50 | -1.47 | 2.48 | 0.61 |
| *Chest/Torso* | 3 (3.5%) | 1.98 | 0.38 | 3.57 | **0.02** | 0.06 | 0.48 | -1.97 | 2.94 | 0.70 |
| *Leg/Foot* | 8 (9.4%) | 0.38 | -0.66 | 1.42 | 0.47 | -0.01 | 0.30 | -0.95 | 1.56 | 0.63 |
| *Arm/Hand* | 75 (88.2%) | -0.57 | -1.51 | 0.36 | 0.23 | 0.01 | *referent* |  | | |
| Region |  | | | | | | | | | |
| *Chittagong* | 15 (17.6%) | 0.94 | 0.17 | 1.71 | **0.02** | 0.05 | 1.30 | 0.03 | 2.57 | **0.045** |
| *Meghna* | 11 (12.9%) | -0.37 | -1.27 | 0.54 | 0.42 | 0.00 | 0.45 | -0.79 | 1.69 | 0.47 |
| *Narayanganj* | 17 (20.0%) | 0.07 | -0.70 | 0.83 | 0.86 | -0.01 | 0.51 | -0.55 | 1.57 | 0.34 |
| *Sreepur* | 42 (49.4%) | -0.42 | -1.02 | 0.18 | 0.17 | 0.01 | *referent* |  | | |
| **Distance of Travel to Nearest Location of Rabies Vax.** |  | | | | | | | | | |
| *≤5 km* | 36 (42.4%) | -0.19 | -0.80 | 0.43 | 0.55 | -0.01 | *referent* |  | | |
| *6-20 km* | 20 (23.5%) | 0.73 | 0.03 | 1.43 | 0.04 | **0.04** | 0.62 | -0.28 | 1.51 | 0.17 |
| *>20 km* | 14 (16.5%) | -0.41 | -1.23 | 0.41 | 0.32 | 0.00 | 0.12 | -0.91 | 1.16 | 0.81 |
| *Unknown* | 15 (17.6%) | -0.20 | -0.99 | 0.60 | 0.63 | -0.01 | -0.13 | -1.13 | 0.88 | 0.80 |
| **Highest Education Level by Household Member** |  | | | | | | | | | |
| *None* | 13 (15.3%) | -0.57 | -1.40 | 0.27 | 0.18 | 0.01 | -0.48 | -1.50 | 0.55 | 0.36 |
| *Primary Education* | 22 (25.9%) | 0.18 | -0.52 | 0.87 | 0.61 | -0.01 | -0.14 | -0.97 | 0.69 | 0.74 |
| *Secondary, College, or Higher*  *Education* | 50 (58.8%) | 0.16 | -0.46 | 0.78 | 0.61 | -0.01 | *referent* |  | | |

^a^ Count (percentage) presented for categorical variables & average presented for continuous variables; total of n=85 canine-bite victims reported within a year prior to the time of survey administration

^b^ Adjusted R-Square = 0.0206; p-value cutoff ≤ 0.05

β values reflect the difference in health seeking behavior score (HSBS)among those with and without the independent variable of interest, controlling for other factors. HSBS ranges from 0 to 5. Bite victims are awarded one point each for: 1) washing of the wound, 2) seeking medical care, 3) initiating PEP, 4) completing PEP, and 5) receiving RIG). Bite victims were assigned half-points for missing response

we did if 'sought medical care' = 0 then all 1) start pep 2) finish pep 3) rig categories = 0, no missings for these 3. they're either Yes/No

if they said yes for medical care then we grade 1/0 not for missings
